# Gillespie-based simulation and inference for non-Markovian stochastic reaction networks

**DOI:** 10.1093/bib/bbag486

**Published:** 2026-09-22

**Authors:** Aurélien Pélissier, Miroslav Phan, Niko Beerenwinkel, María Rodríguez Martínez

**Affiliations:** IBM Research Europe, Säumerstrasse 4, 8803 Rüschlikon, Switzerland; Department of Biosystems Science and Engineering, ETH Zurich, Mattenstrasse 26, 4058 Basel, Switzerland; Institute of Computational Life Sciences, Zürich University of Applied Sciences (ZHAW), Schloss 1, 8820 Wädenswil, Switzerland; IBM Research Europe, Säumerstrasse 4, 8803 Rüschlikon, Switzerland; Department of Biosystems Science and Engineering, ETH Zurich, Mattenstrasse 26, 4058 Basel, Switzerland; Department of Biosystems Science and Engineering, ETH Zurich, Mattenstrasse 26, 4058 Basel, Switzerland; IBM Research Europe, Säumerstrasse 4, 8803 Rüschlikon, Switzerland; Department of Biomedical Informatics and Data Science, Yale School of Medicine, 101 College Street, New Haven, CT 06510, United States

**Keywords:** non-Markovian stochastic simulation, Gillespie algorithm, renewal processes, delayed reactions, systems biology

## Abstract

Discrete stochastic processes are widespread across physics, chemistry, ecology, and beyond. In computational biology and epidemiology, however, most simulators still assume Markovian kinetics with memoryless dynamics, despite growing evidence for history-dependent effects in gene regulation, RNA transcription, cell differentiation, and infection. This reliance on Markovian models limits the routine use and comparison of more realistic, memory-aware descriptions. Here, we develop and benchmark a unified framework for simulating non-Markovian reaction networks using Gillespie-based algorithms. We implement multiple algorithmic classes, including exact, rejection-based, delay-based, and hybrid Markovian/non-Markovian schemes, and compare them across representative biological models. Across three case studies, we show that non-Markovian waiting times can qualitatively change population-level predictions, and that delay-based approximations can break down when intrinsic system timescales approach the imposed delays. We further show how population-level measurements can be used to infer otherwise inaccessible waiting-time distributions, and how non-Markovian structure can be leveraged for sensitivity analysis and statistical inference. To support broad use of these approaches, we release NoMaSS (Non-Markovian Stochastic Simulations), an open-source Python library that provides a unified interface to a wide range of non-Markovian Gillespie algorithms and enables hybrid Markovian/non-Markovian models (https://github.com/AI-SysBio/NoMaSS).

## Graphical Abstract

**Graphical Abstract:**
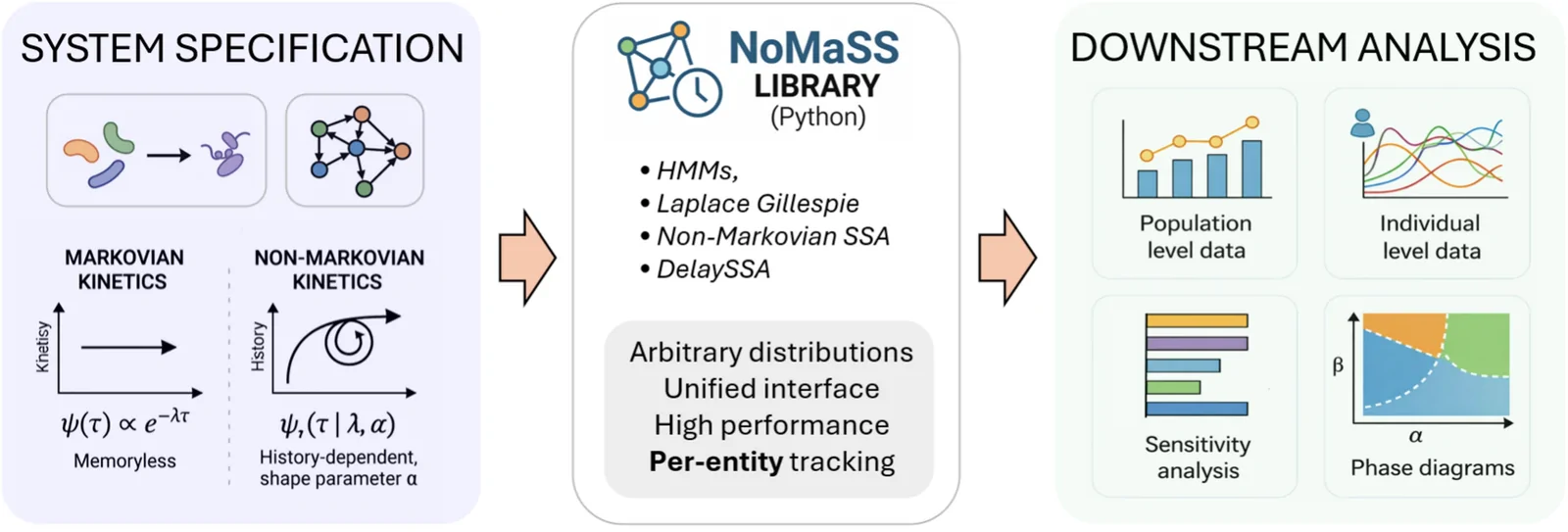

## Introduction

Discrete stochastic processes [1] provide a natural framework for modeling random phenomena in physics [2], biochemistry [3], epidemiology [4], finance [5], and meteorology [6]. In computational biology, they underpin widely used models of gene regulatory networks, signal transduction, and cellular decision-making. To make such systems analytically and numerically tractable, it is common to impose the Markovian assumption, under which the dynamics are memoryless: the probability of future events depends only on the current state and not on how the system arrived there. In continuous time, this implies exponentially distributed inter-event times and, therefore, Poisson-like event statistics, where events occur independently and without intrinsic temporal correlations. This framework leads directly to the classical stochastic Gillespie simulation algorithm (SSA) [7, 8].

Under Markovian kinetics, the Gillespie SSA generates statistically exact realizations of reaction networks with constant reaction propensities and is typically far more efficient than fixed–time-step, agent-based simulations [9]. In its standard formulation, the system’s state $\mathbf{X}(t)$ determines reaction propensities $a_{r}(\mathbf{X}(t))$, which in turn define a total rate $a_{0}$. At each step, the algorithm samples the next reaction time and selects the reaction to fire according to these rates, then updates the state accordingly. This Gillespie SSA has become the workhorse for stochastic modeling in systems and synthetic biology [10–15].

However, many biological systems exhibit pronounced history dependence that violates the Markovian assumption. Hidden molecular states, multi-step biochemical reactions, chromatin remodeling, cell-cycle progression, or refractory periods in neuronal and immune responses all give rise to waiting-time distributions that deviate from the exponential law. In such systems, events are not memoryless: the probability of an event can depend on how long the system has been in its current state, leading to age-dependent or long-tailed waiting times and, consequently, non-Poissonian dynamics. Non-Markovian effects have been documented in a broad range of applications, including quantum devices [16], polymer reactions [17], molecular dynamics [18–20], biochemical reactions in single cells [21, 22], RNA transcription [23], neuronal firing [24], social interactions [25, 26], human activity patterns [27], and earthquakes [28]. In these settings, purely Markovian models can misrepresent variability, temporal correlations, and even qualitative dynamical features.

While analytical approaches can capture certain classes of such non-Markovian effects, especially at steady state [29], in most realistic systems, one must resort to numerical simulation. Over the past two decades, several Gillespie-type strategies have been proposed to accommodate non-exponential inter-event times. Although they share a common “one event at a time” philosophy, they differ substantially in how they encode memory. Broadly, they can be grouped into three classes.

### Class I: Markovian embeddings with hidden or auxiliary states

The first class maintains Markovian dynamics by expanding the state space to include additional variables that account for non-Markovian effects. Hidden Markov models (HMMs) are a discrete-time example: complex temporal dependencies in the observations are captured by Markovian dynamics on a set of hidden states that act as an implicit clock or memory [30, 31]. By chaining multiple hidden states in sequence, such models generate Erlang- or gamma-distributed waiting times. Furthermore, introducing loops or branching between hidden states allows the waiting-time distribution to deviate from simple gamma forms, producing more flexible shapes determined by the transition structure.

In continuous time, a similar idea underlies approaches that represent a non-exponential waiting-time distribution as a combination of exponential phases or as a mixture of reaction rates. The Laplace–Gillespie algorithm [32] makes this explicit by modeling the density of the inter-event-time distribution (IED) as a mixture of exponentials,


(1) \begin{eqnarray*}& \psi(\tau) = \int_{0}^\infty \lambda e^{-\lambda \tau}\,p(\lambda)\,\mathrm{d}\lambda,\end{eqnarray*}

where the rates *λ* can be viewed as latent “tasks” or modes with different priority and *p(λ )* as their distribution. At each SSA step, an effective rate is drawn from *p(λ )* and a standard Gillespie update is performed with these instantaneous rates, yielding exact non-exponential inter-event times while preserving Markovian dynamics in the augmented state space and naturally capturing bursty, priority-queue–like agent behavior.

### Class II: explicit timing via renewal and hazard-based methods

The second class encodes the timing of events directly, without an explicit Markovian assumption and without discretizing the state space into a series of auxiliary stages to mimic memory. Each individual process *i* is treated as a renewal process with a prescribed waiting-time distribution $\psi _{i}(\tau )$, equivalently characterized by its hazard, i.e. its age-dependent instantaneous rate.


\begin{align*} & \lambda_{i}(\tau) = \frac{\psi_{i}(\tau)}{1 - \int_{0}^\tau \psi_{i}(u)\,\mathrm{d}u}. \end{align*}

The non-Markovian Gillespie algorithm (nMGA) [33] and its more efficient variant MOSAIC [34] are representative examples. While the nMGA requires updating the hazards of all reaction channels at every simulation step, MOSAIC utilizes rejection sampling to eliminate these global updates. Both methods track the elapsed time $\tau _{i}$ since the last firing of each process, compute the current hazards $\lambda _{i}(\tau _{i})$, and sample the next event using a Gillespie-based scheme under a short-interval approximation in which hazards are treated as locally constant. The effective step size is controlled internally: smaller time jumps reduce the error introduced by freezing the hazards, at the expense of additional computational cost. MOSAIC adaptively enforces sufficiently small steps so that the remaining approximation error is negligible in practice [34].

Because inter-event time distributions enter explicitly through their hazards, these approaches can accommodate arbitrary waiting-time laws, heterogeneous populations, and couplings to macro-variables, all without time discretization. Their main challenges are algorithmic, namely controlling the per-event computational cost and ensuring that the underlying approximations remain valid in the regimes of interest.

### Class III: hard-coded delayed reactions

The third class introduces non-Markovian effects via hard-coded delayed reactions [35–39]. Namely, when a delayed reaction fires, its effect is not applied immediately; instead, the algorithm samples a delay from a specified distribution and enqueues a completion event (i.e. places it in a queue) to be triggered at a fixed future time. Simulation then proceeds by interleaving two mechanisms: standard Gillespie SSA sampling for instantaneous (Markovian) reactions, and the execution of a priority queue of scheduled completions.

From the perspective of the underlying stochastic model, this hybrid approach is simple, intuitive, and straightforward to implement. However, it departs from the instantaneous-reaction character of classical Gillespie dynamics: once a delayed event is scheduled, it will occur at its assigned completion time, even if the system evolves in ways that would alter or cancel it. Delay-based schemes are therefore exact only under the assumption that the delay distribution remains fixed after initiation and that cancellations are either negligible or explicitly modeled. They also incur memory and sorting overheads when many delayed events are involved.

### Bridging methods and practice

From the perspective of a typical user in bioinformatics or systems biology, the landscape of Gillespie-type extensions for non-Markovian dynamics remains fragmented. Methods are dispersed across communities, implemented in different languages, and often tailored to narrow application domains, which makes them difficult to adopt, combine, and evaluate in a consistent workflow. Existing software such as the DelaySSA toolkit [39] has lowered the barrier to using delayed reactions, but does not natively cover the broader range of Markovian-based memory approximations and hazard-based approaches within a single framework. As a result, practitioners often face a practical bottleneck: deciding which modeling formalism is appropriate for a given biological question, understanding the assumptions that come with each simulator, and integrating memoryless and history-dependent reactions within the same model.

In this article, we bridge this gap by introducing an open-source Python library, NoMaSS, as a computational biology and bioinformatics framework that brings multiple Gillespie-type algorithms for Markovian and non-Markovian processes under a unified interface and supports hybrid reaction networks mixing instantaneous and history-dependent kinetics. The library implements representative methods from the three classes, allows users to specify arbitrary inter-event time distributions, and provides building blocks for downstream analyses commonly needed in practice. We illustrate these capabilities on three biologically motivated case studies, i.e. microbial growth dynamics, stem cell differentiation, and RNA transcription, highlighting workflows such as sensitivity analysis and inference procedures that recover otherwise unmeasurable waiting-time distributions from population-level observations. Alongside this practical guide, we include targeted benchmarks and decision rules to help researchers select, implement, and diagnose non-Markovian simulation approaches in bioinformatics and systems biology.

## NoMaSS: a framework for non-Markovian stochastic simulations

We consider a reaction network composed of reaction channels of the form


\begin{align*} & X \;\xrightarrow{\; r \;}\; P_{1} + \dots + P_{k}, \end{align*}

where *X* denotes one or several reactant species and $P_{1},\dots ,P_{k}$ the products of reaction channel *r*. For each channel *r*, we model the inter-event time *τ* between successive reaction events using a waiting-time distribution


**Figure 1:**
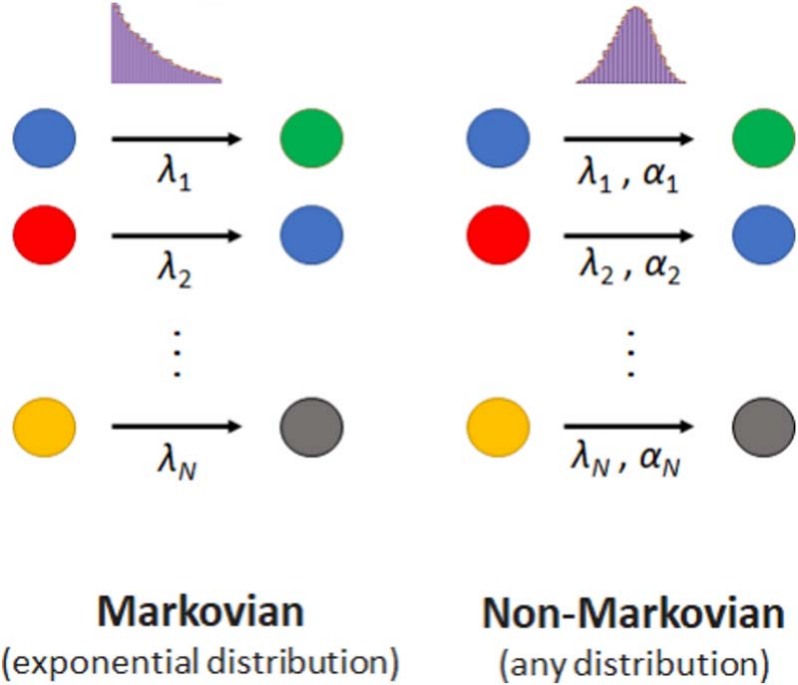
Schematic illustration comparing Markovian reaction dynamics, characterized by constant instantaneous rates and exponentially distributed inter-event times (left), with non-Markovian dynamics governed by generalized waiting-time distributions and time-dependent rates (right). *N*  $\lambda _{i}$, $\lambda _{i}$  $\alpha _{i}$.

\begin{align*} & \psi_{r}(\tau \mid \lambda,\alpha), \end{align*}

parameterized by a nominal rate *λ* and a shape parameter *α*. The rate parameter *λ* sets the characteristic time scale of the reaction. For distributions with a finite mean, we impose $\mathbb{E}[\tau ]=1/\lambda$. In the exponential (Poisson) case, this corresponds to a constant hazard *λ*, recovering the classical Markovian memoryless assumption. For heavy-tailed distributions or distributions with undefined mean, we instead set $\mathrm{median}(\tau )=1/\lambda$, so that *λ* consistently defines a typical time scale across distribution families. The shape parameter *α* controls deviations from exponential kinetics by modulating the variability and tail behavior of $\psi _{r}$. Thus, compared with a standard Gillespie reaction channel, which is typically specified by a single rate parameter, the NoMaSS parameterization usually adds only one additional interpretable shape parameter per non-Markovian reaction channel. For the Gamma and Weibull families, *α =1* recovers exponential waiting times, whereas $\alpha \neq 1$ yields more regular dynamics (narrower distributions) or burstier dynamics (broader distributions). For other families such as Normal, Lognormal, Cauchy, and power-law IEDs, *α* instead tunes dispersion or tail-heaviness without an exponential limit, enabling intrinsically non-exponential waiting times. Figure 1 illustrates this parameterization. Each reaction channel is driven by a rate $\lambda _{i}$, which sets the characteristic time scale, while additional shape parameters $\alpha _{i}$ encode non-Markovian effects.

NoMaSS is a Python library that provides a unified interface to several Gillespie-based approaches for implementing such reaction channels with arbitrary IEDs. At the modeling level, users specify a reaction network, assign a state-dependent IED to each reaction, and choose a simulation scheme for the corresponding subset of reactions. At the algorithmic level, NoMaSS provides a unified API for adding or removing reactions, switching between simulation methods, and combining Markovian and non-Markovian reactions within the same system. In practice, the NoMaSS workflow consists of four main steps: defining the reaction network, assigning an inter-event time distribution to each reaction channel, selecting the appropriate simulation backend, and using the resulting trajectories for benchmarking, parameter inference, sensitivity analysis, or biological interpretation. Furthermore, NoMaSS supports arbitrary IEDs (Table 1) and currently provides four complementary simulation back-ends:

(i) **Laplace–Gillespie.** For reactions whose waiting-time distributions admit a mixture-of-exponentials representation with a monotone decreasing hazard, NoMaSS implements the Laplace–Gillespie scheme [32]. This enables exact simulation of a broad class of long-tailed IEDs by sampling an effective rate from a mixing distribution and then performing a standard Gillespie step. In practice, NoMaSS includes built-in support for common cases such as power-laws ($\propto 1/t^\alpha$), as well as gamma and Weibull IEDs in the decreasing-hazard regime (e.g. gamma with *α <1* and Weibull with *α <1*).
(ii) **MOSAIC.** For arbitrary IEDs specified via hazard functions, NoMaSS includes MOSAIC [34], an optimized variant of the non-Markovian Gillespie algorithm [33]. MOSAIC matches the SSA’s asymptotic complexity and is particularly well-suited when the instantaneous rate remains finite at *τ =0*, such as gamma and Weibull IEDs with $\alpha \ge 1$, as well as log-normal, Pareto, and other commonly used distribution families.
(iii) **Delayed-reaction schemes.** NoMaSS implements Anderson’s *modified next reaction method* [37], which encodes non-Markovianity by scheduling delayed completion events (e.g. in a priority queue) and updating the system state when those events fire. The method can also be combined with other simulation schemes in hybrid models.
(iv) **Hidden Markov embeddings.** While NoMaSS does not provide a dedicated HMM constructor, non-Markovian dynamics can be embedded into a Markovian model by manually introducing intermediate (hidden) states and reactions. This yields a sequence of exponential steps compatible with the classical SSA. In the common special case of a linear chain of identical exponential stages (Erlang waiting times),the same effective waiting-time law can be represented more compactly within MOSAIC by using a gamma IED with an integer shape parameter.

**Table 1:** Summary of supported inter-event distributions (IEDs) for NoMaSS back-end methods, parameterized by rate and shape parameters *λ*  *α* characterizing deviations from Markovian dynamics (full parameterizations and implementation details are provided in *α*  Tables 2 and 3

PDF name	DelaySSA	MOSAIC	Laplace
Exponential	✓	✓	✓
Gamma	✓	✓ ($\alpha \ge 1$)	✓ ($\alpha \leq 1$)
Weibull	✓	✓ ($\alpha \ge 1$)	✓ ($\alpha \leq 1$)
Gaussian / Normal	✓	✓	*×*
Lognormal	✓	✓	*×*
Cauchy	✓	✓	*×*
Power-law (Pareto)	✓	✓	*×*
Power-law (Lomax)	✓	✓	✓

All methods share a common internal representation of reactions, state variables, and random number generation, making it straightforward to switch between algorithms or to simulate the same model under different assumptions about the inter-event time distributions. Users can select the simulation scheme on a per-reaction basis, enabling fine-grained control over which parts of the network are treated as Markovian, renewal-based, or delay-based.

### Toy-model benchmark: unconstrained symmetric population growth

**Figure 2:**
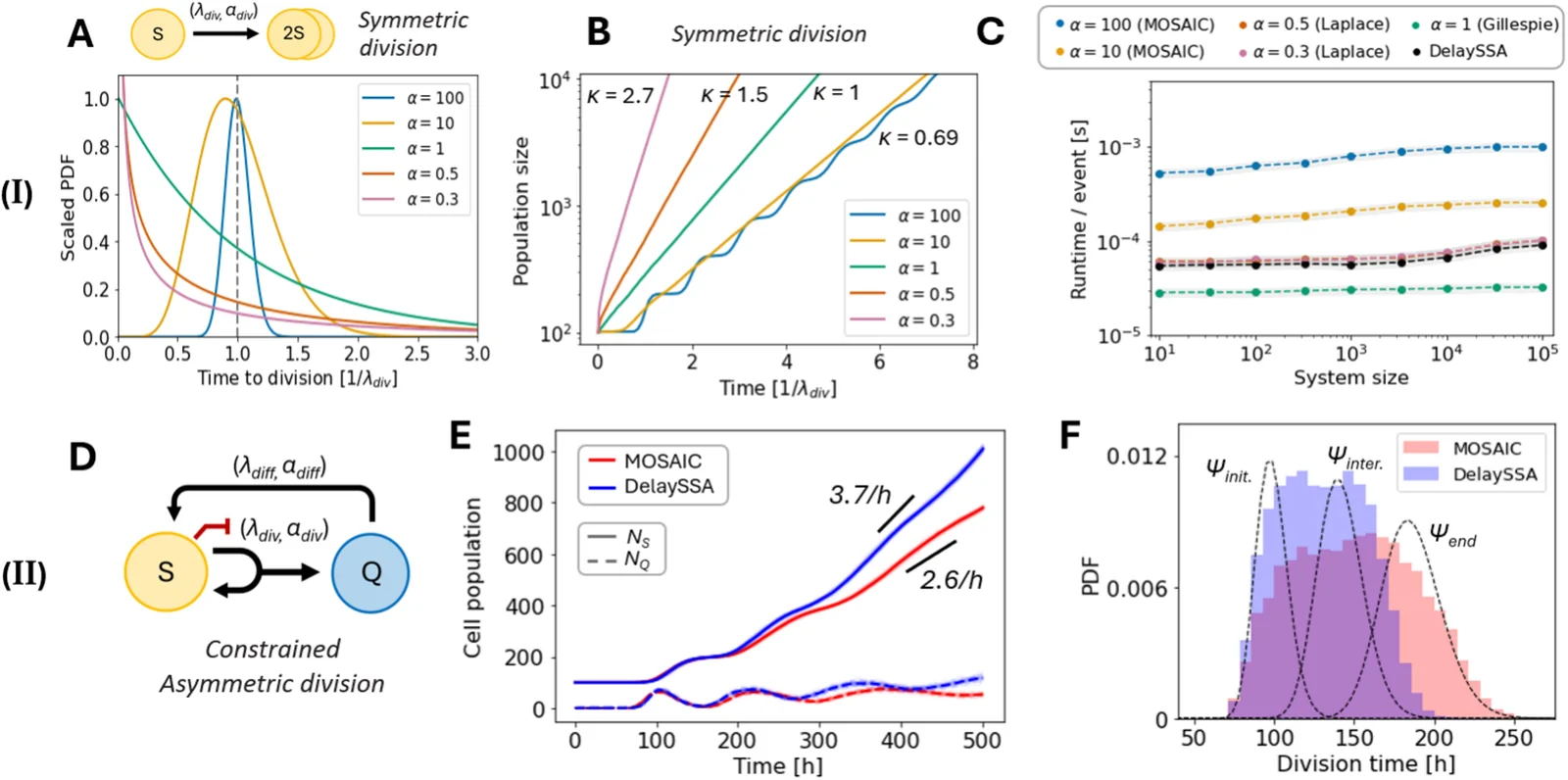
Dynamics of (I) symmetric and (II) asymmetric growth under non-Markovian division. (A) Top: schematic of unconstrained symmetric division (*S → 2S*). Bottom: gamma-distributed interdivision times for different shape parameters *α*, with each probability density function (PDF) scaled to unit maximum for visual clarity. (B) Simulated population growth from 100 initial reactants undergoing unconstrained symmetric division (*S → 2S*) with gamma-distributed interdivision times at fixed mean division time and varying *α*. Corresponding fitted growth rates (*κ*) are shown for reference. (C) Per-event computational cost versus system size for different methods implemented in NoMaSS for the same unconstrained symmetric division process (*S → 2S*). Shaded bands denote the standard deviation across simulation replicates. (D) Schematic of crowd-limited microbial growth in *Caulobacter crescentus*: a stalked cell (*S*) divides into a new stalked cell and a motile, quiescent cell (*Q*), which cannot divide but can differentiate into a stalked cell. Increasing cell density reduces the division rate. (E) Population dynamics starting from 100 stalked cells ($1/\lambda _{\mathrm{div,0}} = 71.6$, $1/\lambda _{\mathrm{diff}} = 28.7$, *K = 500*), averaged over 50 simulations, simulated using the MOSAIC and DelaySSA backends of NoMaSS. Asymptotic linear growth-rate fits are shown for reference. We observe a divergence in asymptotic growth rates, where DelaySSA overestimates the population growth by failing to account for increasing density during the division waiting period. (F) Density histograms of aggregated stalked-cell division-time distributions for both methods. Dashed curves represent the effective IEDs calculated by evaluating $\lambda _{\mathrm{div}}(N)$ at the population counts corresponding to the beginning, middle, and end of the simulation (Equation (3)). These curves illustrate the shift toward longer inter-division times as growth becomes constrained.

As an initial benchmark, we consider a simple pure-birth process $S \xrightarrow{} 2S$, where each particle divides independently with a gamma-distributed IED of mean $\mu =1/\lambda _{\mathrm{div}}$ and shape *α* (Fig. 2A). Analytically, the population grows exponentially as $N(t) \propto e^{\kappa t}$, where the effective growth rate *κ* is sensitive to the variance in division timing. Even when the mean division time *μ* is held constant, *κ* depends on the shape parameter *α* (Fig. 2B, Supplementary A.2) according to:


(2) \begin{eqnarray*}& \kappa \;=\; \frac{\alpha\big(2^{1/\alpha}-1\big)}{\mu}.\end{eqnarray*}

This relationship captures the transition between two physical extremes. In the Markovian limit (*α = 1*), the memoryless nature of the exponential distribution yields a simple growth rate $\kappa = 1/\mu$. Conversely, as division times become more regular ($\alpha \to \infty$), the process becomes deterministic; the population doubles exactly every *μ* units of time, resulting in a slower asymptotic growth rate $\kappa = (\ln 2)/\mu$. Physically, this behavior arises because heavy-tailed distributions (*α < 1*) allow a fraction of “fast-dividers” to dominate the population expansion early on, disproportionately accelerating the total growth. In contrast, more regular, “clock-like” division times (*α> 1*) lack these early events, leading to a systematic reduction in the observed growth rate. This example illustrates how neglecting non-Markovian IEDs can systematically bias inferred kinetic parameters from population-level data.

Beyond the physical dynamics, the choice of IED shape parameter significantly impacts the numerical efficiency of the simulation. As shown in Fig. 2C, the computational cost per event varies across the different backends implemented in NoMaSS. As expected, classical SSA is fastest because it requires neither rejection sampling nor queue management. Laplace-Gillespie and DelaySSA show comparable performance across shape parameters, reflecting their similar tree-based bookkeeping, i.e. rates in the former and scheduled completion times in the latter. In contrast, MOSAIC’s runtime depends strongly on the IED through the rejection rate. For gamma IEDs, the expected number of rejections increases approximately linearly with *α* (Table 3), so narrower distributions lead to higher computational cost.

Overall, for IEDs that cannot be handled efficiently by the Laplace–Gillespie method, DelaySSA is typically the fastest option. This advantage, however, relies on the delay-based assumption that an event’s completion time is fixed at initiation, i.e. the IED does not change after the event has been scheduled. This assumption holds for the present renewal division process but may be violated in models in which hazards depend on evolving state variables. In such cases, MOSAIC provides the more appropriate simulation scheme.

## Use cases of NoMaSS in real biological systems

### Application I: When delay-based approaches fail in state-dependent microbial growth

We now consider the population dynamics of the bacterium *Caulobacter crescentus* [40], which divides asymmetrically into a replication-competent stalked cell (*S*) and a motile quiescent cell (*Q*) that does not divide immediately but can later differentiate into a stalked cell (Fig. 2D). Before a stalked cell can divide again, it must complete several prerequisite steps, including DNA replication, chromosome segregation, and physical separation [41], and therefore, division cannot occur immediately after birth. Hence, an exponential waiting-time model, which assigns the highest division probability at *t=0*, is physiologically implausible. More generally, any model with a nonzero instantaneous division rate immediately after cell birth cannot capture the refractory period inherent to the cell cycle.

To represent the true division dynamics, we use single-cell division-time measurements for stalked cells under dilute (i.e. unconstrained, low-density) conditions [40] to calibrate the baseline inter-division-time distribution. Among the candidate distributions tested, a log-normal IED provided the best fit (Supplementary A.1, Table S1), capturing the delayed onset of division and the observed variability across individual cells. We then embed this empirically calibrated distribution into a state-dependent population growth model, in which increasing crowding slows division through a smooth density-dependent modulation of the division rate:

- **Stalked-cell asymmetric division:**  *S → S + Q*, with inter-division times drawn from a log-normal distribution. The intrinsic mean division rate $\lambda _{\mathrm{div},0}$ and shape parameter $\alpha _{\mathrm{div}}$ are obtained from fits to dilute conditions [40]. To model growth limitation, we introduce a crowding factor *ϕ (N) = 1 + N/K*, where $N = N_{S} + N_{Q}$ is the total population of stalked ($N_{S}$) and quiescent ($N_{Q}$) cells, and *K* is a carrying-capacity constant. The effective division rate is modulated as
  (3) \begin{eqnarray*}& \lambda_{\mathrm{div}}(N) \;=\; \frac{\lambda_{\mathrm{div},0}}{\phi(N)},\end{eqnarray*}
  ensuring that division slows as the system becomes crowded. This formulation implements *throughput-limited* dynamics: while growth is exponential at low density (*N ≪ K*), the total population flux $J = N \lambda _{\mathrm{div}}(N)$ saturates at $\lambda _{\mathrm{div},0} K$ as $N \to \infty$. Consequently, the population size increases approximately linearly in the high-density regime, reflecting a constant production rate limited by available surface area or nutrients.
- **Quiescent-cell differentiation:**  *Q → S*, with non-Markovian differentiation dynamics. Because direct measurements of $Q\!\to \!S$ waiting times are experimentally challenging [42], we model differentiation using a log-normal IED with mean $1/\lambda _{\mathrm{diff}}$, which we constrain from steady-state stalked-to-quiescent population ratios [40]. For simplicity, we set the log-normal shape parameter to match that of the division process, ensuring that the relative variance in timing is consistent across all cellular transitions.

In Supplementary A.3, we show how varying the shape parameters influences the system’s growth rate and how the model’s transient oscillations dampen over time, with broader IEDs reducing their amplitude. We also note that in the regime $\lambda _{\mathrm{diff}} \gg \lambda _{\mathrm{div}}$, the system simplifies to symmetric population growth.

This setting exposes a sharp difference between exact hazard-based simulation and delay-based approximations. Crucially, in MOSAIC non-Markovian reaction times are generated *from the current hazard* and therefore respond to changes in the system state throughout the waiting period. By contrast, in a standard DelaySSA, each division (or differentiation) event is assigned a completion time *once at initiation* and is then executed at that scheduled time, even if the environment changes in the meantime. When the rates vary slowly compared with the typical delay, this approximation is accurate; however, when the modulation is fast on the scale of the IED, DelaySSA can become strongly biased.

As illustrated in Fig. 2E, our simulation reveals a regime where state-dependent growth inhibition unfolds on a timescale commensurate with the baseline cellular division time. This alignment arises because the physical drivers of crowding—the division events themselves—dictate the rate of environmental decay. Consequently, the inter-division delay is not a static parameter but a dynamic state-variable that evolves alongside the population (Fig. 2F). Given a mean division time of *∼*150 h within a total observation window of 500 h, the system operates in a transient state where the *memory* of the environment is refreshed at a rate nearly identical to the underlying biological frequency.

In this setting, MOSAIC and DelaySSA rapidly diverge. DelaySSA systematically overestimates the number of completed divisions because many events remain “locked in” based on the favorable conditions at initiation, failing to account for the increasing system density during the waiting period (Fig. 2F). This discrepancy is not merely transient: in the crowded regime, it produces an effective growth rate that significantly differs from the MOSAIC results (Fig. 2E; 3.7/h for DelaySSA versus 2.6/h for MOSAIC).

We note that MOSAIC provides a ground truth in this state-dependent context, because it is a hazard-based rejection sampling scheme designed for the statistically exact realization of time-varying propensities. Its precision is limited only by the user-defined rejection bound, and we empirically confirmed numerical convergence by observing that predicted growth rates remained invariant under increasingly stringent rejection frequencies. Consequently, while the fixed-delay approximation in DelaySSA breaks down under rapid environmental modulation, MOSAIC remains robustly responsive to state-dependent feedback throughout the inter-event interval.

Overall, this example highlights a key modeling message: delay-based schemes are exact for renewal reactions with fixed post-initiation delays, but can be unreliable when inter-event statistics depend on evolving state variables (density, nutrients, stress, etc.). NoMaSS makes this distinction explicit: DelaySSA remains a convenient and often fast choice when its assumptions are satisfied, while MOSAIC provides a near exact hazard-based alternative when reaction rates must remain responsive to a changing environment.

### Application II: inference of unmeasurable differentiation times from stem cell population data

Another interesting application for NoMaSS is stem cell differentiation, the process by which specialized cells are formed from stem cells [43]. Stochastic fluctuations in key transcription factors play important roles in the early stages of differentiation [44], but it is less clear whether progression along a differentiation trajectory is itself intrinsically stochastic or reflects a tightly orchestrated sequence of intermediate meta-stable states. The latter hypothesis could explain the apparently precise regulation of differentiation at the single-cell level.

From a modeling point of view, transitions between functional cell types can be described as a stochastic process with *memory*, where “memory” reflects how long a cell has resided in its current state before progressing. In practice, however, waiting-time distributions for individual cells are rarely observable: what is typically measured are population fractions, i.e. the proportions of cells in each subpopulation at a set of time points. Here, we use NoMaSS to ask whether a previously proposed HMM for stem cell differentiation is consistent with the data [22], and to infer the underlying single-cell waiting-time distributions directly from population dynamics.

Concretely, we consider the stochastic differentiation of mouse embryonic stem cells (ESC) along the neural lineage. The experimental data used for this application consist of the population fractions of three functional cell stages, embryonic stem cells (ESC), epiblast-like (EPI) states, and neuroprogenitor cells (NPC), measured at seven time points over a duration of *∼*200 h [22]. The original study modeled these population dynamics using an HMM [30], in which each observable cell type (ESC, EPI, and NPC) is associated with multiple hidden microstates. Fitting the experimental data required 19 or 20 such microstates, depending on the cell line. Although this formulation is flexible, it introduces a large number of parameters (roughly one per microstate), and different combinations of transition and emission rates can lead to similar fits. Importantly, under the simplifying assumption that all transitions between microstates occur at the same rate, the HMM reduces to an Erlang (gamma) waiting-time distribution for each functional transition. In other words, the HMM encodes an implicit hypothesis about the single-cell IED. With NoMaSS, we can test this hypothesis directly while working with a much simpler model.

**Figure 3:**
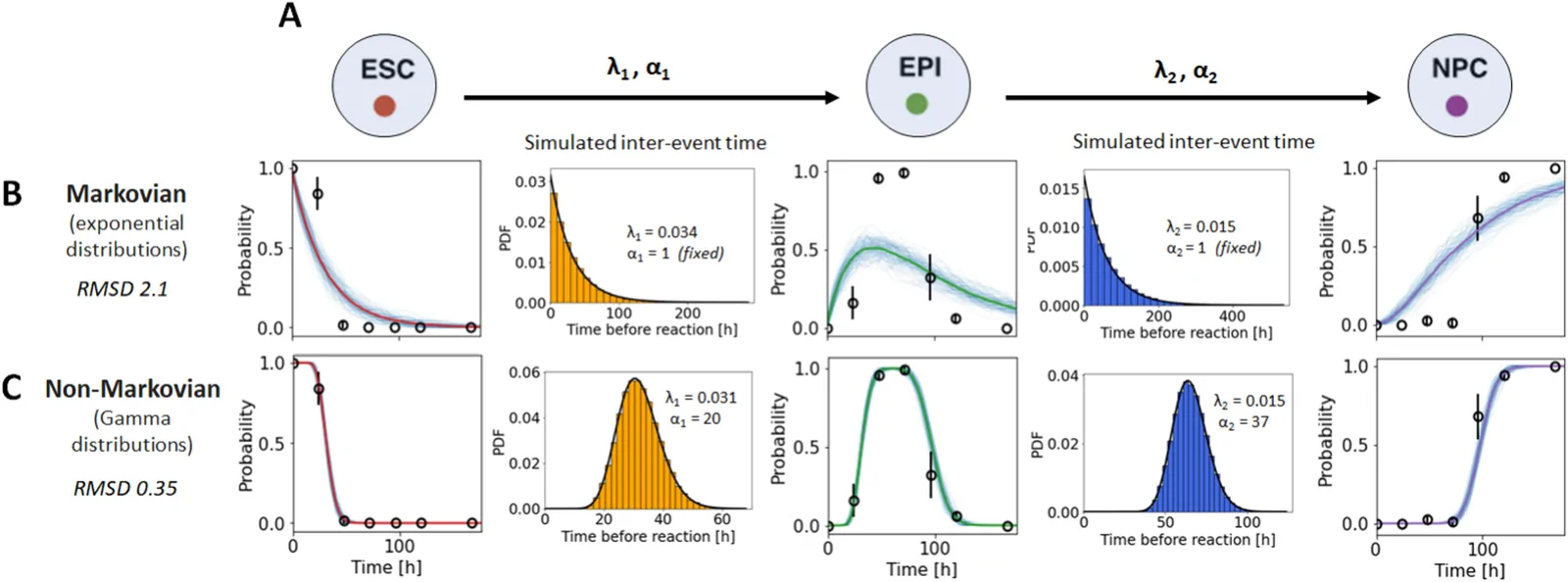
Inference of non-Markovian stem cell differentiation dynamics. (A) Mouse stem cells at three stages of differentiation. From the ESC stage, cells may differentiate into EPI, which in turn may differentiate into NPC. The inter-event times associated with these two differentiation steps are modeled using either (B) exponential or (C) gamma distributions. The average RMSDs between simulated and measured data at each time point and for each cell type are indicated for both models. The Markovian case is fitted by fixing the shape parameters $\alpha _{1} = \alpha _{2} = 1$ (exponential inter-event times). Circles and error bars represent the mean and standard deviation of the stem cell populations measured at different time points [22]; solid lines show population dynamics obtained by averaging 1000 NoMaSS simulations with parameters that minimize the RMSD. Individual simulations are shown as narrow lines. The inferred inter-event time distributions after parameter optimization are plotted for the ESC*→*EPI and EPI*→*NPC transitions, respectively. Here, peaks at *t=0*, failing to capture the biological lag or refractory period represented by the gamma distributions.

In NoMaSS, we represent the differentiation process using only two stochastic reactions (Fig. 3):

- **ESC *→* EPI**, with inter-event times drawn from a distribution $\psi _{1}(\tau \mid \lambda _{1},\alpha _{1})$,
- **EPI *→* NPC**, with inter-event times drawn from $\psi _{2}(\tau \mid \lambda _{2},\alpha _{2})$.

Because these inter-event time distributions are assumed to depend only on the current cell type and not on global population variables, a delay-based scheme is appropriate: in NoMaSS we therefore use a DelaySSA back-end, in which each differentiation event is scheduled at commitment and completed after a random delay drawn from $\psi _{r}$. This directly matches the biological assumption that once a cell has committed to differentiate, its completion time is not influenced by subsequent changes in the rest of the population.

We first compare a Markovian model to a non-Markovian gamma model. In the Markovian case, both transitions are exponential, i.e. we fix $\alpha _{1} = \alpha _{2} = 1$ and infer rates $\lambda _{1},\lambda _{2}$. In the gamma case, we infer $(\lambda _{1},\alpha _{1})$ and $(\lambda _{2},\alpha _{2})$ from the population fractions of ESC, EPI and NPC over time by minimizing the root mean square deviation (RMSD) between data and simulations. Averaging 1000 NoMaSS trajectories for each parameter set, we obtain an RMSD of 2.1 for the exponential (Markovian) model and 0.35 for the gamma (non-Markovian) model, i.e. an order-of-magnitude improvement. This difference is clearly visible in Fig. 3, where only the gamma-based Non-Markovian model reproduces the timing and magnitude of the observed population changes.

We then repeat the analysis using other waiting-time families for $\psi _{1}$ and $\psi _{2}$, including normal, log-normal and Weibull distributions (Supplementary B). Briefly, the Weibull model performs substantially worse (RMSD = 0.69) than gamma (0.35), normal (0.38) and log-normal (0.39) models, which all give similarly good fits. This discrepancy arises from the Weibull distribution’s polynomial increasing hazard rate; its characteristic “short tail” fails to capture the sustained stochasticity of the system and over-penalizes longer waiting times, which does not accurately reflect the underlying biological dynamics. We also examined the impact of preprocessing by fitting both the raw proportion data and a logit-transformed version (to spread points close to 0 and 1). For logit-transformed data, the gamma distribution yields the lowest RMSD; for non-transformed data, the log-normal distribution is marginally better. In both cases, however, gamma, normal, and log-normal distributions capture the data comparably well, consistent with their similar shapes in the parameter regime here inferred (Fig. S1).

Finally, a gamma distribution with integer shape parameter *k* is equivalent to the sum of *k* exponential waiting times (an Erlang distribution). Such a distribution naturally arises when a process progresses through a sequence of hidden intermediate states. The good fit of an Erlang-type waiting-time model is therefore consistent with earlier HMM-based interpretations of stem cell differentiation as a multi-stage process [22]. Using NoMaSS, we can represent this behavior directly with a compact non-Markovian model and compare it with alternative waiting-time assumptions.

### Application III: quantifying the impact of non-Markovian dynamics on observed mRNA counts

We next model transcription, the process by which DNA is copied into messenger RNA (mRNA). After RNA polymerase binds to the promoter region, a nascent RNA molecule is elongated until the full gene has been transcribed; the resulting mRNA can then be translated into protein and is eventually degraded at an approximately constant rate. Real-time measurements in single cells have revealed pronounced stochasticity in transcription [45], motivating a large body of stochastic models for gene expression [11, 23, 46].

A widely used description is the two-state or telegraph model, in which the promoter switches between an ON and an OFF state at constant rates. Transcription can occur only in the ON state, leading to bursty, non-Poissonian mRNA fluctuations that match many experimental observations [47]. This model has been studied extensively [48] and used to infer kinetic parameters for thousands of genes from single-cell RNA-seq data [49, 50]. Nevertheless, there is clear evidence that the statistics of molecular fluctuations often deviate from simple Markovian predictions [23, 51], implying that non-exponential waiting times, and thus non-Markovian descriptions, are required. This is supported by the discovery of biological *refractory periods* and non-Poissonian noise profiles that cannot be captured by constant transition rates.

**Figure 4:**
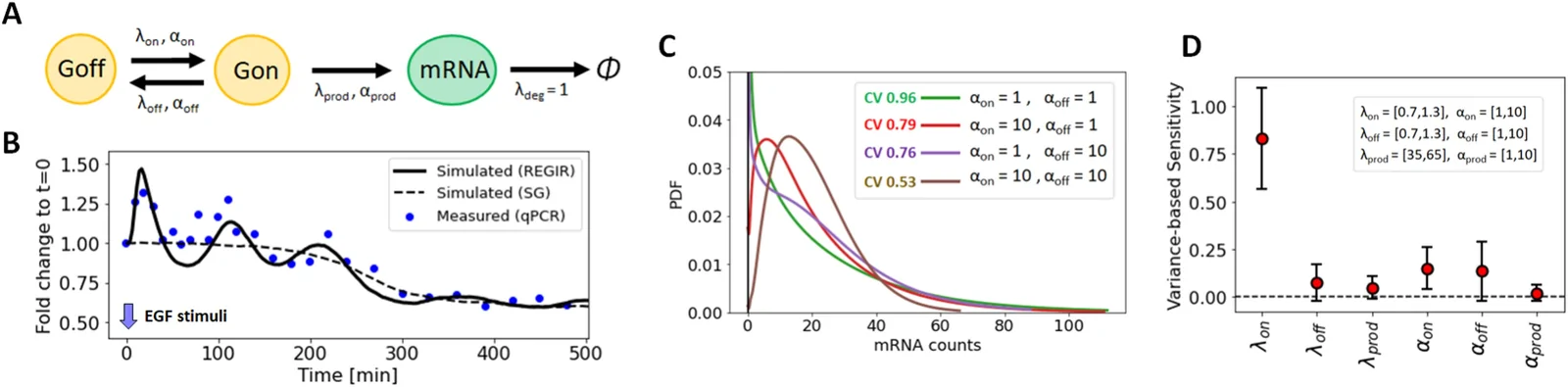
Non-Markovian transcription dynamics and their impact on mRNA variability. (A) Cartoon of the two-state model of RNA transcription with non-Markovian dynamics. The gene promoter switches between ON and OFF; mRNA is produced only in the ON state and degrades at a constant rate. (B) Average mRNA profile of the gene SLC7A5 after stimulation by epidermal growth factor (EGF) [53], measured by quantitative PCR (qPCR) and normalized to 1 at the time of stimulation. Simulated populations are averaged over 10k trajectories for both the Markovian model (SSA, RMSD = 0.016) and the non-Markovian model (NoMaSS, RMSD = 0.0074), using the best-fit parameters for each. (C) Steady-state mRNA distributions for different shape-parameter combinations, with $\lambda _{\mathrm{on}} = 1$, $\lambda _{\mathrm{off}} = 3$, $\lambda _{\mathrm{prod}} = 100$ and $\alpha _{\mathrm{prod}} = 1$ fixed. Densities are estimated by Gaussian kernel density estimation after a log transform (see Materials and methods); the coefficient of variation (CV) is reported for each case. (D) Sobol variance-based sensitivity analysis of the CV of the steady-state mRNA distribution generated by the two-state model. The grid comprises 128 points per parameter. The rate $\lambda _{\mathrm{on}}$, which controls promoter activation, has the strongest influence on the variance.

NoMaSS enables us to probe directly how non-Markovian features of the underlying molecular steps influence observable mRNA distributions. To do so, we model RNA transcription using four reactions (Fig. 4A):

- **Promoter activation and deactivation:**  $\mathrm{G}_{\mathrm{off}} \xrightarrow{\lambda _{\mathrm{on}},\ \alpha _{\mathrm{on}}} \mathrm{G}_{\mathrm{on}}$ and $\mathrm{G}_{\mathrm{on}} \xrightarrow{\lambda _{\mathrm{off}},\ \alpha _{\mathrm{off}}} \mathrm{G}_{\mathrm{off}}$. The promoter switches between OFF and ON, with inter-event times following gamma distributions with parameters $(\lambda _{\mathrm{on}},\alpha _{\mathrm{on}})$ and $(\lambda _{\mathrm{off}},\alpha _{\mathrm{off}})$, respectively. These non-exponential kinetics account for the fact that promoter switching typically involves multiple sequential molecular steps, such as chromatin remodeling or transcription factor assembly, whose convolution yields gamma-like statistics.
- **RNA production:**  $\mathrm{G}_{\mathrm{on}}\xrightarrow{\lambda _{\mathrm{prod}},\ \alpha _{\mathrm{prod}}} \mathrm{G}_{\mathrm{on}} + \mathrm{mRNA}$. When the promoter is ON, mRNA production (initiation and elongation) is modeled by a gamma waiting-time distribution with parameters $(\lambda _{\mathrm{prod}},\alpha _{\mathrm{prod}})$. This represents the cumulative time required for initiation, promoter escape, and elongation—a multi-step process that often exhibits a significant lag compared with memoryless production.
- **RNA degradation:**  $\mathrm{mRNA} \xrightarrow{\lambda _{\mathrm{deg}}} \varnothing$. In contrast, mRNA decay is modeled with exponential waiting times. As a single-particle, first-order process where degradation is equally likely at any time regardless of the molecule’s age, it is well described by memoryless kinetics [52]

Without loss of generality, all rates are expressed in units of $\lambda _{\mathrm{deg}}$ (i.e. $\lambda _{\mathrm{deg}}=1$), following standard practice [48, 49].

In this application, a delay-based implementation is appropriate because we do not explicitly model energy-limited elongation or other time-dependent mechanisms that render elongation rates strongly state- or history-dependent. Prior work has identified regimes in which such energy-limited effects necessitate fully renewal/hazard-based simulation (e.g. MOSAIC) [33, 34]; those conditions are not required here.

To highlight the role of non-Markovian dynamics, we use time-resolved mRNA profiles measured by qPCR after EGF stimulation [53]. Several genes (e.g. CREG1, DDB2, SLC7A5, NFYC, NR4A1, and PTGS2) display oscillatory responses that cannot be reproduced by the Markovian two-state model (Fig. 4B and Supplementary Section C.2). In contrast, after fitting the gamma shape parameters $(\alpha _{\mathrm{on}},\alpha _{\mathrm{off}},\alpha _{\mathrm{prod}})$ with NoMaSS, the non-Markovian model can reproduce both the period and the decaying amplitude of these oscillations. The oscillation period *T* is largely determined by the mean switching times, $T \approx 1/\lambda _{\mathrm{on}} + 1/\lambda _{\mathrm{off}}$, while the damping of the oscillations is controlled by the shape parameters $\alpha _{\mathrm{on}}$ and $\alpha _{\mathrm{off}}$ (Supplementary Section C.1). This pattern, seen across multiple genes [53], supports the view that promoter switching is not well described by purely Markovian kinetics [54].

Beyond fitting specific time courses, NoMaSS makes it straightforward to explore how non-Markovian features shape the steady-state distribution of mRNA counts. In the bursty regime, roughly, $\lambda _{\mathrm{off}}> \lambda _{\mathrm{on}}$ and $\lambda _{\mathrm{prod}} \gg \lambda _{\mathrm{on}},\lambda _{\mathrm{off}}$ [23], the shape parameters $\alpha _{\mathrm{on}}$ and $\alpha _{\mathrm{off}}$ have a strong impact on the distribution. For example, in Fig. 4C the Markovian case ($\alpha _{\mathrm{on}} = \alpha _{\mathrm{off}} = 1$) yields a high probability of observing zero mRNAs, whereas increasing both shape parameters to 10 makes zero counts highly unlikely, even though the mean rates are unchanged. This illustrates the value of a framework that enables the systematic exploration of both Markovian and non-Markovian regimes within a single unified modeling environment.

To systematically quantify the parameters primarily driving variability in mRNA counts, we performed a Sobol variance-based global sensitivity analysis [55]. Unlike local sensitivity methods that evaluate effects at a single point in the parameter space, Sobol analysis decomposes the total output variance into contributions from individual parameters and their nonlinear interactions. This approach provides a comprehensive mapping of how parametric uncertainty propagates to the system’s output. Because the steady-state mean of the mRNA distribution depends solely on mean waiting times and remains independent of the shape parameters [56] (Fig. S8), we utilized the coefficient of variation (${\mathrm{CV}} = \sigma /\mu$) as our primary output metric (Fig. 4D). Our analysis reveals that the ${\mathrm{CV}}$ is relatively robust to parameters governing mRNA production ($\lambda _{\mathrm{prod}}$, $\alpha _{\mathrm{prod}}$). This aligns with prior analytical work demonstrating that the distribution shape is insensitive to production variations within the regime $\lambda _{\mathrm{prod}} \gg \lambda _{\mathrm{on}}, \lambda _{\mathrm{off}}$ [48, 56]. In contrast, the system exhibits high sensitivity to switching kinetics: $\lambda _{\mathrm{on}}$ exerts a significantly stronger influence on the ${\text{ CV}}$ than $\lambda _{\mathrm{off}}$, whereas the shape parameters $\alpha _{\mathrm{on}}$ and $\alpha _{\mathrm{off}}$ contribute with comparable magnitudes. These trends are further supported by phase diagrams (Fig. S9), which illustrate how varying shape parameters affect the ${\text{ CV}}$ landscape.

Together, these results demonstrate how NoMaSS can be used to (i) fit non-Markovian transcription models to time-course data, (ii) quantify the effect of non-exponential waiting times on steady-state mRNA distributions, and (iii) perform phase-diagram and sensitivity analyses across wide parameter ranges. This is particularly important in gene expression, where kinetic parameters span orders of magnitude [49] and non-Markovian effects can significantly distort observable mRNA statistics.

## Discussion

Non-Markovian effects are increasingly recognized as a key feature of biological stochastic dynamics, yet they remain underused in everyday modeling because they substantially complicate simulation, calibration, and model comparison. Here we take a deliberately practical approach: we organize major Gillespie-type methods for non-Markovian dynamics into a small set of conceptual families, benchmark their assumptions and performance on representative biological problems, and make them accessible through a unified software interface (NoMaSS) that supports hybrid Markovian/non-Markovian systems.

A central design choice is the parameterization of waiting-time distributions as $\psi _{r}(\tau \mid \lambda ,\alpha )$, where *λ* governs the mean timescale and *α* captures deviations from exponential, memoryless kinetics, such as increased regularity or burstiness. Across all applications, changing *α* while keeping the mean waiting time $\mathbb{E}[\tau ]$ fixed led to substantial shifts in biological observables, including growth rates, oscillation amplitudes, and steady-state variability.

This added flexibility should nevertheless be carefully assessed. In noisy or sparse biological datasets, non-Markovian waiting-time distributions may improve the fit by replicating technical noise, hidden heterogeneity, or outliers rather than true biological memory. We therefore recommend using the Markovian Gillespie model as the baseline and retaining non-Markovian structure only when the improvements are robust across model comparisons, held-out data, alternative waiting-time families, or sensitivity analyses. Because the Markovian case is recovered for common distributions such as Gamma and Weibull when *α = 1*, this comparison is straightforward within the NoMaSS framework. NoMaSS provides a high-performance implementation that serves as a foundation for extensive downstream analyses. Its efficiency facilitates external computational pipelines—such as sensitivity analysis, e.g. Sobol indices, and phase-diagram across wide parameter ranges—that require high-throughput ensemble simulation. Simultaneously, NoMaSS supports mechanistic, event-driven studies by tracking per-entity ages and gathering individual-level statistics, providing the granular data necessary for rigorous parameter inference.

Our three case studies illustrate complementary ways in which memory shapes biological inference and prediction. In microbial growth, realistic division and differentiation time distributions changed both the effective growth rate and the degree of transient synchronization compared with mean-matched Markovian models. This example also clarifies when delay-based implementations fail: if propensities evolve on timescales comparable with the inter-event times, scheduled delays no longer reflect the current system state. In stem cell differentiation, we showed that a high-dimensional HMM can be collapsed into a compact non-Markovian description by replacing latent progression with a gamma/Erlang inter-event distribution while still reproducing population-level dynamics. Because NoMaSS makes the Erlang–HMM correspondence explicit, comparisons with alternative waiting-time assumptions (normal, log-normal, and Weibull) become transparent: differences in fit reflect distributional shape rather than differing numbers of latent states. In RNA transcription, non-exponential promoter switching altered both transient responses and steady-state mRNA distributions. Combining global sensitivity analysis with phase diagrams further revealed which kinetic rates and shape parameters control these effects, highlighting how memory in promoter dynamics propagates to observable mRNA variability.

A consistent takeaway is that delay-based methods provide a simple and often effective default for many biological systems [39], while retaining flexibility in the choice of IED. When delays are specified at reaction initiation and remain invariant to later state changes, delay schemes integrate naturally with the standard SSA. They are typically more efficient than non-Markovian Gillespie variants because simulation reduces to standard event selection plus a delay queue, avoiding rejection or resampling to handle competing non-exponential waiting times. This makes them well suited for common systems-biology motifs such as maturation, transport, and processing steps.

Delay formulations are not universal. When propensities or waiting-time laws depend strongly on the evolving state, so that “commitment” at initiation becomes outdated, or when delays are frequently interrupted, canceled, or rescheduled, both their conceptual validity and computational advantages can break down [34]. In such regimes, instantaneous renewal formulations that continuously update hazards based on current age and state can provide a more faithful representation and may even reduce bookkeeping overhead [12, 13, 34].

Among approaches that represent non-Markovian dynamics through equivalent Markovian formulations, the Laplace–Gillespie method occupies a more specialized niche, in which waiting-time distributions are represented as mixtures of exponentials. Many biological mechanisms that generate non-Markovian dynamics, such as maturation or refractory effects, suppress very short waiting times. In contrast, the places substantial probability mass near zero [32]. This mismatch makes it less well suited for several biochemical and cellular examples considered here. Nevertheless, it remains valuable in domains characterized by strong heterogeneity in effective rates, such as earthquakes [57], epidemics [58], and bursty human interaction processes [59, 60].

From a computational perspective, all event-driven Gillespie variants can become slow for very large reaction networks or under extreme time-scale separation, where rare events are selected infrequently and steady state is reached only after long runtimes. In NoMaSS, this limitation follows from the design: because we track individual ages or initiation times and accumulate per-reactant statistics, runtime increasingly becomes dominated by bookkeeping and propensity evaluation as system size grows. Consequently, NoMaSS is not intended as a high-throughput engine for massive particle pools. Instead, it is optimized for settings where individual-level timing information is biologically meaningful and where non-Markovian effects are expected to matter.

In genuinely large-scale regimes, coarse-grained abstractions are often preferable [61, 62]. When many reactants are statistically similar, it is often unnecessary to keep track of every individual waiting time. Instead, reactants can be grouped into *bins* based on their internal *age* (i.e. the time elapsed since a reactant’s last event), with hazards and event counts updated at the bin level. Such grouped-clock representations reduce memory and scheduling overhead while preserving the key distributional effects of non-exponential waiting times. They also reflect a broader statistical principle: as group sizes increase, individual stochastic fluctuations average out and the dynamics approach a continuous (concentration-like) description, whereas fluctuations in small or critical subpopulations can dominate system-level behavior. In this large-scale limit, hybrid approximations become a natural extension, particularly piecewise deterministic Markov process–style schemes that treat large, fast components through deterministic continuous dynamics while retaining discrete stochasticity for rare events or small-count species [63–65]. Similar hybrid modeling strategies are implemented in platforms such as COPASI, which supports deterministic, stochastic, and hybrid deterministic–stochastic simulations of biochemical networks [66].

Finally, non-Markovian settings involving interactions between multiple reactants, most notably processes on temporal networks [67], remain comparatively less developed. A central difficulty is that the notion of an inter-event time is not uniquely defined. Waiting times may be assigned to edges (pairwise contact renewal), nodes (activity renewal), or higher-order interaction motifs, and each choice leads to a different hazard structure and corresponding simulation algorithm [34, 59]. This ambiguity helps explain why much of the temporal-network literature has focused on activity-driven models, which define node-level activation processes and generate contacts through pairing rules rather than modeling interaction events directly as competing hazards in continuous time [68]. Only recently have Gillespie-style methods been extended to accommodate non-Markovian contact processes on networks [34, 59, 69]. Systematic connections between activity-driven models and hazard-based event simulation, particularly for epidemiological dynamics on time-varying contact networks, therefore remain an open and practically important direction.

Overall, our results point to a clear modeling strategy. Begin with delay-based formulations when biological processes involve well-defined delays that remain stable after initiation. When reaction timings depend on the evolving state of the system or when events can be interrupted or rescheduled, hazard-based renewal methods provide a more faithful description. Finally, when non-exponential waiting times arise from sequences of hidden intermediate steps, they can often be represented through Markovian embeddings, allowing simulation with standard Gillespie dynamics. By placing these alternatives behind a common interface, NoMaSS enables systematic comparison of modeling assumptions and elevates memory to a testable dimension of stochastic models that can be evaluated, reported, and reproduced alongside structural and parametric assumptions.

## Materials and methods

**Code and data availability.** The NoMaSS implementation, input data, and scripts required to reproduce the analyses and figures presented in this article are publicly available on GitHub at https://github.com/AI-SysBio/NoMaSS.

### Parameterization of IEDs implemented in NoMaSS

NoMaSS implements a set of commonly used IEDs to model non-Markovian reaction dynamics, including Weibull, Gamma, Normal, Lognormal, Cauchy, and two power-law families (Pareto I and Lomax/Pareto II). For consistency across models and with prior work in stochastic simulation, each IED is specified by two parameters: a nominal rate *λ* and a dimensionless shape parameter *α* (Table 2).

**Table 2:** Parameterization of IEDs implemented in NoMaSS.

IED name	PDF parameterization (nominal rate $\boldsymbol\lambda$ and shape parameter $\boldsymbol\alpha$)
Exponential	$\psi (\tau )=\lambda e^{-\lambda \tau }$
Weibull	$\beta =\alpha \left (\lambda \,\Gamma (\tfrac{\alpha +1}{\alpha })\right )^{\alpha }, \quad \psi (\tau )=\beta \,\tau ^{\alpha -1}\exp \!\left (-\tfrac{\beta }{\alpha }\tau ^{\alpha }\right )$
Gamma	$\beta =\alpha \lambda , \quad \psi (\tau )=\dfrac{\beta ^{\alpha }}{\Gamma (\alpha )}\,\tau ^{\alpha -1}e^{-\beta \tau }$
Gaussian/Normal	$\mu =\lambda ^{-1},\ \sigma =\alpha \mu , \quad \psi (\tau )=\dfrac{1}{\sigma \sqrt{2\pi }}\exp \!\left (-\tfrac{(\tau -\mu )^{2}}{2\sigma ^{2}}\right )$
Lognormal	$\mu = \log \left (\frac{1}{ \lambda \sqrt{1 + \alpha ^{2}}}\right ),\ \sigma = \sqrt{\log \left (1+ \alpha ^{2}\right )}, \quad \psi (\tau )=\dfrac{1}{\tau \sigma \sqrt{2\pi }}\exp \!\left (-\tfrac{(\ln \tau -\mu )^{2}}{2\sigma ^{2}}\right )$
Cauchy	$\mu =\lambda ^{-1},\ \gamma =\alpha \mu , \quad \psi (\tau )=\dfrac{1}{\pi \gamma \left (1+\big (\tfrac{\tau -\mu }{\gamma }\big )^{2}\right )}$
Power-law (Pareto I)	$\tau _{0}=\lambda^{-1}2^{-1 / \alpha }, \quad \psi (\tau)= \begin{cases} \alpha\,\tau_0{^{\alpha}}\, \tau^{-(\alpha+1)}, \quad \tau \ge \tau_0, \\ 0, \quad \tau < \tau_0.\end{cases}$
Power-law (Lomax/Pareto II)	$\kappa =(2^{1/\alpha }-1)\lambda , \quad \psi (\tau )=\alpha \kappa \,(1+\kappa \tau )^{-(\alpha +1)}$

Note: Each PDF is specified by a nominal rate *λ* and a shape parameter *α*. For distributions with a finite mean, *λ* is chosen such that $\mathbb{E}[\tau ]=1/\lambda$. For heavy-tailed distributions without a well-defined mean (Cauchy and the two power-law cases), *λ* is set via the median, i.e. $\mathrm{median}(\tau )=1/\lambda$. The shape parameter *α* is dimensionless (scale-free): for Normal, Lognormal, and Cauchy it equals the CV ($\alpha =\sigma /\mathbb{E}[\tau ]$), while for Gamma, Weibull and the power-law families it matches the standard shape/tail exponent.

**Table 3:** Hazard-based and Laplace-mixture ingredients used by NoMaSS.

IED name	Instantaneous rate $\boldsymbol\lambda (\boldsymbol\tau )$	Max rate $\boldsymbol\lambda$ _max_	MOSAIC [34] implementation	Laplace mixture $\boldsymbol{p(\ell )}$ [32] such that $\boldsymbol\psi (\boldsymbol\tau ) = \int _{0}^\infty \ell e^{-\ell \tau }\,p(\ell )\,\mathrm{d}\ell$
Exponential	$\lambda (\tau )=\lambda$	*λ*	fixed	$p(\ell ) = \delta (\ell -\lambda )$
Weibull	$\lambda (\tau )=\beta \,\tau ^{\alpha -1}$	*∞*	$\lambda (\tau _{\max }) \ [\alpha \ge 1]$	*ℓ* is positive *α*-stable [70, 71] with $\mathbb{E}[e^{-\ell \tau }]=e^{-(\mu \tau )^{\alpha }},\ \mu =\left (\frac{\beta }{\alpha }\right )^{1/\alpha }$ *[α < 1]*
Gamma	$\lambda (\tau )=\frac{\beta ^{\alpha }\,\tau ^{\alpha -1}e^{-\beta \tau }}{\Gamma (\alpha ,\beta \tau )}$	$\beta \ [\alpha \ge 1]$	$\lambda (\tau _{\max }) \ [\alpha \ge 1]$	$p(\ell )=\frac{\beta ^{\alpha }\,H(\ell -\beta )} {\Gamma (\alpha )\Gamma (1-\alpha )\,\ell \,(\ell -\beta )^{\alpha }} \ [\alpha < 1]$
Gaussian/Normal	$\lambda (\tau ) = \frac{\psi (\tau )}{S(\tau )}$	*∞*	$\lambda (\tau _{\max })$	*×*
Lognormal	$\lambda (\tau ) = \frac{\psi (\tau )}{S(\tau )}$	finite	fixed	*×*
Cauchy	$\lambda (\tau ) = \frac{\psi (\tau )}{S(\tau )}$	finite	fixed	*×*
Power-law (Pareto)	$\lambda(\tau)= \begin{cases} \frac{\alpha}{\tau}, \quad \tau \ge \tau_0\\ 0, \quad \tau < \tau_0 \end{cases}$	$\frac{\alpha }{\tau _{0}}$	fixed	*×*
Power-law (Lomax)	$\lambda (\tau )=\frac{\alpha \kappa }{1+\kappa \tau }$	$\alpha \kappa$	fixed	$p(\ell )=\frac{\ell ^{\alpha -1}e^{-\ell /\kappa }}{\Gamma (\alpha )\kappa ^{\alpha }}$

Note: For each supported IED with density $\psi (\tau )$, we report the instantaneous rate (hazard) $\lambda (\tau )=\psi (\tau )/S(\tau )$, where the survival function is $S(\tau )=\int _{\tau }^{\infty }\psi (u)\,du$. When a simple closed form is available, we list it explicitly; otherwise we retain the generic ratio form $\psi (\tau )/S(\tau )$ (see Table 2 for complete parameterization). We also report the maximal hazard $\lambda _{\max }$ used to set the rejection/thinning bound in MOSAIC. Here $\tau _{\max }$ denotes the current age of the *oldest* reactant (i.e. the one that has not reacted for the longest time), at which the bound is evaluated. When available, we provide the Laplace-mixture density *p(ℓ )* such that $\psi (\tau )=\int _{0}^{\infty }\ell e^{-\ell \tau }\,p(\ell )\,d\ell$, enabling simulation via the Laplace–Gillespie construction. We use *ℓ* for the latent mixture rate to avoid confusion with the nominal rate parameter *λ*.

The nominal rate *λ* sets the characteristic time scale. When the mean inter-event time is finite, we choose parameters such that $\mathbb{E}[\tau ]=\frac{1}{\lambda }$. For heavy-tailed distributions whose mean may be undefined (Cauchy and the two power-law cases), we instead fix the time scale using the median, $\mathrm{median}(\tau )=\frac{1}{\lambda }$. In addition to setting the typical time scale, *λ* also coincides with the *mean observed instantaneous rate* of the renewal process: if one samples the system at a uniformly random time, the average observed rate over this observation distribution follows $\mathbb{E}_{\mathrm{obs}}[\lambda (\tau )]=1/\mathbb{E}[\tau ]=\lambda$ whenever the mean exists [34].

The shape parameter *α* controls the deviation from memoryless (exponential) kinetics by tuning dispersion and/or tail behavior, and is defined to be scale-free across all families. For Cauchy, Normal, and Lognormal IEDs we set *α* to the CV, $\alpha = \mathrm{std}(\tau ) / \mathbb{E}[\tau ],$ so that *α* directly measures relative variability. For Gamma and the power-law families, *α* coincides with the standard shape/tail exponent used in the usual parameterization.

### MOSAIC and Laplace Gillespie implementation in NoMaSS

Table 3 summarizes the operational quantities required by the simulation backends implemented in NoMaSS, i.e. the MOSAIC renewal scheduler [34] and the Laplace–Gillespie construction [32]. Both approaches rely on the same age-dependent propensity, given by the instantaneous rate (hazard) $\lambda (\tau )=\psi (\tau )/S(\tau )$, but use it in different ways.

The Laplace–Gillespie backend applies when the waiting-time law admits an exponential-mixture representation. In that case one can introduce a latent rate *ℓ* drawn from *p(ℓ )* and simulate conditionally Markovian dynamics between renewal updates, which can yield substantial speedups when many processes share the same mixture structure.

In MOSAIC, hazards are evaluated at the current “age” of each pending event and used within a rejection/thinning procedure; efficiency is controlled by an upper bound $\lambda _{\max }$ on the hazard. For IED families with bounded hazards, NoMaSS uses the corresponding finite $\lambda _{\max }$ directly. For families whose hazards can grow without bound, NoMaSS tracks the largest current age among pending processes ($\tau _{\max }$). The operative bound is then taken as $\lambda (\tau _{\max })$, so the rate bound automatically tracks the oldest active renewal clock in the system. In addition, NoMaSS enforces the MOSAIC accuracy criterion


\begin{align*} & N_{r}\,\lambda_{\max} \;\ge\; 20\,\lambda_{r} \qquad \text{for all channels}\ r, \end{align*}

where $N_{r}$ is the number of currently eligible renewals/reactants in channel *r* and $\lambda _{r}$ is the nominal rate of that channel. This requirement makes the thinning bound sufficiently conservative, effectively capping the MOSAIC step size and rendering the approximation error negligible in practice, as recommended in [34].

#### Earth mover distance

Also known as the Wasserstein or Kantorovich metric [72], the Earth mover distance (EMD) [73] is a measure of the distance between two probability distributions $P = [p_{i}]$ and $Q = [q_{j}]$ (*i,j*: index of instances in the probability distribution) over a metric space *D*. Where $D = [d_{i,j}]$ is the ground distance between clusters $[p_{i}]$ and $[q_{j}]$. EMD reflects the minimal amount of *work* that must be used to transform one distribution into the other by moving *distribution mass* around. To compute EMD, we first need to find the optimal flow $F = [f_{i,j}]$ to minimize the overall cost when moving from *P* to *Q*.


(4) \begin{eqnarray*}& \min \sum_{i=1}^{n} \sum_{j=1}^{m} f_{i,j} d_{i,j}\end{eqnarray*}

Finding the optimal flow *F* is typically related to an instance of the transportation problem, and can be efficiently solved in polynomial time [74]. EMD is then defined as the minimal work normalized by the total flow:


(5) \begin{eqnarray*}& \mathrm{EMD}(P,Q) = \frac{\sum_{i=1}^{n} \sum_{j=1}^{m} f_{i,j} d_{i,j}}{\sum_{i=1}^{n} \sum_{j=1}^{m} f_{i,j}}\end{eqnarray*}

Note that EMD satisfies the metric properties, i.e. positivity, symmetricity, triangle inequality, and free from any choice of bin size if $d_{\mathrm{i,j}}$ follows the metric properties

In our application, the EMD between IEDs is quantified in unit of time. However, to ensure consistency of the EMD value over different parameter space, we normalize the distance matrix $[d_{i,j}]$ by the mean inter event time ($1/\lambda _{0}$) of the reaction channel. We use the Fast EMD python implementation [75] to efficiently compute the distances. The distances are measured by sampling $1 \times 10^4$ points from both the theoretical and simulated inter-event distributions, which result in an intrinsic EMD on the order of 1% (Fig. S10). This comes from the fact that sampled points from equivalent distributions will not be strictly equal, which will result in a non-zero EMD.

### Parameter optimization

For each NoMaSS simulation, we quantify its *error* by comparing the simulated population dynamics to the experimentally measured populations. We define the *score* of the model as the RMSD between the measured and simulated population averaged over all timepoints and reactants. In the case of the stem cell differentiation, the populations describe proportions bounded between 0 and 1. This may bias the optimization towards ignoring variations close to very low (*∼ 0*) and very high (*∼ 1*) values, as a proportion of 98% will be considered closer to 99% than 50% is to 52%. To correct for this bias, we transform both simulated and measured populations with an inverse sigmoid function. For experimental reasons, proportions lower than *1%* or higher than *99%* are considered not reliable enough, so we only apply the transformation within that range:


(6) \begin{eqnarray*}& \begin{aligned} &y_{\mathrm{new}} =\log \left(\frac{y}{1-y}\right) \ \ \mathrm{for} \ \ 1\% \leq y \leq 99\%\\ &y_{\mathrm{new}} = 4.6 \ \ \mathrm{for} \ \ y> 99\%\\ &y_{\mathrm{new}} = -4.6 \ \ \mathrm{for} \ \ y < 1\%\\ \end{aligned}\end{eqnarray*}

Then, the score function is minimized with the maxLIPO algorithm [76], which is both parameter free and provably better than a random search. It is a good alternative to Bayesian optimization methods [77], that typically require the definition of prior assumptions about the function being optimized and thus require domain knowledge.

### Density estimation of mRNA distribution

Generally, considering a kernel density estimator for a random variable *Y=g(X)* denoted $f_{Y}(y)$, one can retrieve the density function of *X*, denoted $f_{X}(x)$, with the relation (up to a scaling factor) [78]


(7) \begin{eqnarray*}& f_{X}(x) \sim f_{Y}(y)\left|\frac{d}{d x} g(x)\right|.\end{eqnarray*}

Since in our problem, the mRNA density count is bounded between *[0,+∞ ]*, we cannot estimate the density directly with a gaussian kernel as it may infer non zero density for negative mRNA counts. Thus, we first transform mRNA counts with the function *y = g(x) = łog (x)* so that the transformed data are unbounded ($[-\infty ,+\infty ]$). Then, we retrieve the density of the mRNA counts with


\begin{align*} & f_{X}(x) \sim \frac{f_{Y}(\log (x))}{x}. \end{align*}

### Variance-based sensitivity analysis

A variance-based sensitivity analysis decomposes the variance of the output of the model $\operatorname{Var}(Y)$ into fractions which can be attributed to inputs $\{S_{i}\}$, where a first-order sensitivity index is defined as


\begin{align*} & S_{i} = \frac{V_{i}}{\operatorname{Var}(Y)}, \end{align*}

We note that with this definition, the sum of the sensitivities over all input parameters is always one $\left ( \sum S_{i} = 1\right )$. We estimate sensitivity indices numerically with a quasi-Monte Carlo method, where points are sampled with the Sobol quasi-random sequences [55]. The python library Salib [79] is used to perform this analysis.

**Key Points**

- We present NoMaSS, an open-source Python framework that unifies Gillespie-based algorithms for simulating Markovian, non-Markovian, and hybrid stochastic reaction networks.
- Across three biological case studies, we show that non-Markovian waiting-time distributions can qualitatively alter model predictions, including population dynamics, variability, and inferred system behavior.
- We benchmark exact, rejection-based, delay-based, and hybrid simulation schemes, clarifying when different non-Markovian algorithms are accurate, efficient, or prone to approximation error.
- We show that delay-based approximations can fail when intrinsic system timescales interact with imposed delays, highlighting the need for flexible hazard- and renewal-based formulations.
- We demonstrate how population-level measurements can be used to infer otherwise inaccessible waiting-time distributions and support sensitivity analysis in non-Markovian biological models.

## Supplementary Material

SI_bbag486

## Code availability

NoMaSS is implemented as a python library (-pip install NoMaSS), and its source code is accessible at https://github.com/AI-SysBio/NoMaSS. All datasets used in this studies are made available in the repository .

## References

[1] Ross SM . Stochastic Processes, Vol. 2. New York: Wiley, 1996.

[2] Van Kampen, N. G. (1992). Stochastic Processes in Physics and Chemistry (Vol. 1). Amsterdam: Elsevier.

[3] Cao Y, Samuels DC. Discrete stochastic simulation methods for chemically reacting systems. *Methods Enzymol* 2009;454:115–40. 10.1016/S0076-6879(08)03805-6 PMC3492891 19216925

[4] Yan P. Distribution theory, stochastic processes and infectious disease modelling. In: Brauer F, van den Driessche P, Wu J (eds). Mathematical Epidemiology, pp. 229–93. Berlin, Heidelberg: Springer, 2008.

[5] Kijima M. Stochastic Processes with Applications to Finance. Boca Raton: Chapman and Hall/CRC, 2002.

[6] Tseng Y-T, Kawashima S, Kobayashi S et al. Forecasting the seasonal pollen index by using a hidden Markov model combining meteorological and biological factors. *Sci Total Environ* 2020;698:134246. 10.1016/j.scitotenv.2019.134246 31505344

[7] Gillespie DT . A general method for numerically simulating the stochastic time evolution of coupled chemical reactions. *J Comput Phys* 1976;22:403–34.

[8] Gillespie DT . Exact stochastic simulation of coupled chemical reactions. *J Phys Chem* 1977;81:2340–61.

[9] Figueredo GP, Siebers P-O, Owen MR et al. Comparing stochastic differential equations and agent-based modelling and simulation for early-stage cancer. *PLoS One* 2014;9:e95150. 10.1371/journal.pone.0095150 PMC3994035 24752131

[10] Arkin A, Ross J, McAdams HH. Stochastic kinetic analysis of developmental pathway bifurcation in phage *λ*-infected *Escherichia coli* cells. *Genetics* 1998;149:1633–48. 10.1093/genetics/149.4.1633 PMC1460268 9691025

[11] Martinez MR, Soriano J, Tlusty T et al. Messenger RNA fluctuations and regulatory RNAs shape the dynamics of a negative feedback loop. *Phys Rev E* 2010;81:031924. 10.1103/PhysRevE.81.031924 20365787

[12] Thomas MJ, Klein U, Lygeros J et al. A probabilistic model of the germinal center reaction. *Front Immunol* 2019;10:689. 10.3389/fimmu.2019.00689 PMC6456718 31001283

[13] Pélissier A, Akrout Y, Jahn K et al. Computational model reveals a stochastic mechanism behind germinal center clonal bursts. *Cells* 2020;9:1448. 10.3390/cells9061448 PMC7349200 32532145

[14] Vestergaard CL, Génois M. Temporal Gillespie algorithm: fast simulation of contagion processes on time-varying networks. *PLoS Comput Biol* 2015;11:10. 10.1371/journal.pcbi.1004579 PMC4627738 26517860

[15] Sun L, Ashcroft P, Ackermann M et al. Stochastic gene expression influences the selection of antibiotic resistance mutations. *Mol Biol Evol* 2020;37:58–70. 10.1093/molbev/msz199 PMC6984361 31504754

[16] White GAL, Hill CD, Pollock FA et al. Demonstration of non-Markovian process characterisation and control on a quantum processor. *Nat Commun* 2020;11:1–10. 10.1038/s41467-020-20113-3 PMC7725842 33298929

[17] Guérin T, Bénichou O, Voituriez R. Non-Markovian polymer reaction kinetics. *Nat Chem* 2012;4:568–73. 10.1038/nchem.1378 22717443

[18] Ayaz C, Tepper L, Brünig FN et al. Non-Markovian modeling of protein folding. *Proc Natl Acad Sci* 2021;118:e2023856118. 10.1073/pnas.2023856118 PMC8346879 34326249

[19] Vroylandt H, Goudenège L, Monmarché P et al. Likelihood-based non-Markovian models from molecular dynamics. *Proc Natl Acad Sci* 2022;119:e2117586119. 10.1073/pnas.2117586119 PMC9060509 35320038

[20] Lickert B, Stock G. Modeling non-Markovian data using Markov state and Langevin models. *J Chem Phys* 2020;153:244112. 10.1063/5.0031979 33380115

[21] Bratsun D, Volfson D, Tsimring LS et al. Delay-induced stochastic oscillations in gene regulation. *Proc Natl Acad Sci* 2005;102:14593–8. 10.1073/pnas.0503858102 PMC1253555 16199522

[22] Stumpf PS, Smith RCG, Lenz M et al. Stem cell differentiation as a non-Markov stochastic process. *Cell Syst* 2017;5:268–282.e7. 10.1016/j.cels.2017.08.009 PMC5624514 28957659

[23] Cao Z, Grima R. Analytical distributions for detailed models of stochastic gene expression in eukaryotic cells. *Proc Natl Acad Sci* 2020;117:4682–92. 10.1073/pnas.1910888117 PMC7060679 32071224

[24] Baddeley R, Abbott LF, Booth MCA et al. Responses of neurons in primary and inferior temporal visual cortices to natural scenes. *Proc R Soc Lond Ser B Biol Sci* 1997;264:1775–83. 10.1098/rspb.1997.0246 PMC1688734 9447735

[25] Jiang Z-Q, Xie W-J, Li M-X et al. Calling patterns in human communication dynamics. *Proc Natl Acad Sci* 2013;110:1600–5. 10.1073/pnas.1220433110 PMC3562842 23319645

[26] Zhao K, Bianconi G. Social interactions model and adaptability of human behavior. *Front Physiol* 2011;2:101. 10.3389/fphys.2011.00101 PMC3243102 22194724

[27] Barabasi A-L . The origin of bursts and heavy tails in human dynamics. *Nature* 2005;435:207–11. 10.1038/nature03459 15889093

[28] Corral A . Long-term clustering, scaling, and universality in the temporal occurrence of earthquakes. *Phys Rev Lett* 2004;92:108501. 10.1103/PhysRevLett.92.108501 15089251

[29] Zhang J, Zhou T. Markovian approaches to modeling intracellular reaction processes with molecular memory. *Proc Natl Acad Sci* 2019;116:23542–50. 10.1073/pnas.1913926116 PMC6876203 31685609

[30] Rabiner L, Juang B. An introduction to hidden Markov models. *IEEE ASSP Mag* 1986;3:4–16.

[31] Yoon B-J . Hidden Markov models and their applications in biological sequence analysis. *Curr Genomics* 2009;10:402–15. 10.2174/138920209789177575 PMC2766791 20190955

[32] Masuda N, Rocha LEC. A Gillespie algorithm for non-Markovian stochastic processes. *SIAM Rev* 2018;60:95–115.

[33] Boguná M, Lafuerza LF, Toral R et al. Simulating non-Markovian stochastic processes. *Phys Rev E* 2014;90:042108. 10.1103/PhysRevE.90.042108 25375439

[34] Pélissier A, Phan M, le Bail D et al. Unifying non-Markovian dynamics and agent heterogeneity in scalable stochastic networks. Nat Commun 2026;17:1–16. 10.1038/s41467-026-69817-y PMC13066373 41771859

[35] Barrio M, Burrage K, Leier A et al. Oscillatory regulation of Hes1: discrete stochastic delay modelling and simulation. *PLoS Comput Biol* 2006;2:e117. 10.1371/journal.pcbi.0020117 PMC1560403 16965175

[36] Cai X . Exact stochastic simulation of coupled chemical reactions with delays. *J Chem Phys* 2007;126:12. 10.1063/1.2710253 17411109

[37] Anderson DF . A modified next reaction method for simulating chemical systems with time dependent propensities and delays. *J Chem Phys* 2007;127:21. 10.1063/1.2799998 18067349

[38] Ramaswamy R, Sbalzarini IF. A partial-propensity formulation of the stochastic simulation algorithm for chemical reaction networks with delays. *J Chem Phys* 2011;134:1. 10.1063/1.3521496 21218996

[39] Xiaoming F, Zhou X, Gu D et al. DelaySSAToolkit. Jl: stochastic simulation of reaction systems with time delays in Julia. *Bioinformatics* 2022;38:4243–5. 10.1093/bioinformatics/btac472 35799359

[40] Iyer-Biswas S, Wright CS, Henry JT et al. Scaling laws governing stochastic growth and division of single bacterial cells. *Proc Natl Acad Sci* 2014;111:15912–7. 10.1073/pnas.1403232111 PMC4234605 25349411

[41] Wang JD, Levin PA. Metabolism, cell growth and the bacterial cell cycle. *Nat Rev Microbiol* 2009;7:822–7. 10.1038/nrmicro2202 PMC2887316 19806155

[42] England JC, Perchuk BS, Laub MT et al. Global regulation of gene expression and cell differentiation in *Caulobacter crescentus* in response to nutrient availability. *J Bacteriol* 2010;192:819–33. 10.1128/JB.01240-09 PMC2812448 19948804

[43] Alvarado AS, Yamanaka S. Rethinking differentiation: stem cells, regeneration, and plasticity. *Cell* 2014;157:110–9. 10.1016/j.cell.2014.02.041 PMC4074550 24679530

[44] Chambers I, Silva J, Colby D et al. Nanog safeguards pluripotency and mediates germline development. *Nature* 2007;450:1230–4. 10.1038/nature06403 18097409

[45] Elowitz MB, Levine AJ, Siggia ED et al. Stochastic gene expression in a single cell. *Science* 2002;297:1183–6. 10.1126/science.1070919 12183631

[46] Shahrezaei V, Swain PS. Analytical distributions for stochastic gene expression. *Proc Natl Acad Sci* 2008;105:17256–61. 10.1073/pnas.0803850105 PMC2582303 18988743

[47] Suter DM, Molina N, Gatfield D et al. Mammalian genes are transcribed with widely different bursting kinetics. *Science* 2011;332:472–4. 10.1126/science.1198817 21415320

[48] Iyer-Biswas S, Hayot F, Jayaprakash C. Stochasticity of gene products from transcriptional pulsing. *Phys Rev E* 2009;79:031911. 10.1103/PhysRevE.79.031911 19391975

[49] Larsson AJM, Johnsson P, Hagemann-Jensen M et al. Genomic encoding of transcriptional burst kinetics. *Nature* 2019;565:251–4. 10.1038/s41586-018-0836-1 PMC7610481 30602787

[50] Kim JK, Marioni JC. Inferring the kinetics of stochastic gene expression from single-cell RNA-sequencing data. *Genome Biol* 2013;14:1–12. 10.1186/gb-2013-14-1-r7 PMC3663116 23360624

[51] Park SJ, Song S, Yang G-S et al. The chemical fluctuation theorem governing gene expression. *Nat Commun* 2018;9:297. 10.1038/s41467-017-02737-0 PMC5775451 29352116

[52] Wang Y, Liu CL, Storey JD et al. Precision and functional specificity in mRNA decay. *Proc Natl Acad Sci* 2002;99:5860–5. 10.1073/pnas.092538799 PMC122867 11972065

[53] Zeisel A, Köstler WJ, Molotski N et al. Coupled pre-mRNA and mRNA dynamics unveil operational strategies underlying transcriptional responses to stimuli. *Mol Syst Biol* 2011;7:529. 10.1038/msb.2011.62 PMC3202801 21915116

[54] Molina N, Suter DM, Cannavo R et al. Stimulus-induced modulation of transcriptional bursting in a single mammalian gene. *Proc Natl Acad Sci* 2013;110:20563–8. 10.1073/pnas.1312310110 PMC3870742 24297917

[55] Sobol IM . Global sensitivity indices for nonlinear mathematical models and their Monte Carlo estimates. *Math Comput Simul* 2001;55:271–80.

[56] Wang Z, Zhang Z, Zhou T. Analytical results for non-Markovian models of bursty gene expression. *Phys Rev E* 2020;101:052406. 10.1103/PhysRevE.101.052406 32575237

[57] Saichev A, Sornette D. Theory of earthquake recurrence times. J Geophys Res Solid Earth 2007;112:B04313 (pp. 1–26).

[58] Vazquez A, Rácz B, Lukács A et al. Impact of non-Poissonian activity patterns on spreading processes. *Phys Rev Lett* 2007;98:158702. 10.1103/PhysRevLett.98.158702 17501392

[59] Sheng A, Su Q, Li A et al. Constructing temporal networks with bursty activity patterns. *Nat Commun* 2023;14:7311. 10.1038/s41467-023-42868-1 PMC10640578 37951967

[60] Takaguchi T, Masuda N. Voter model with non-Poissonian interevent intervals. *Phys Rev E Stat Nonlin Soft Matter Phys* 2011;84:036115. 10.1103/PhysRevE.84.036115 22060464

[61] Sanft KR, Othmer HG. Constant-complexity stochastic simulation algorithm with optimal binning. *J Chem Phys* 2015;143:08B609_1. 10.1063/1.4928635 PMC4545075 26298116

[62] Gil J, Polański A. Application of Gillespie algorithm for simulating evolution of fitness of microbial population. *Appl Comput Sci* 2022;18:5–15.

[63] Herbach U, Bonnaffoux A, Espinasse T et al. Inferring gene regulatory networks from single-cell data: a mechanistic approach. *BMC Syst Biol* 2017;11:1–15. 10.1186/s12918-017-0487-0 PMC5697158 29157246

[64] Boxma O, Kaspi H, Kella O et al. On/off storage systems with state-dependent input, output, and switching rates. *Probab Eng Inf Sci* 2005;19:1–14. 10.1017/S0269964805050011

[65] Pakdaman K, Thieullen M, Wainrib G. Fluid limit theorems for stochastic hybrid systems with application to neuron models. *Adv Appl Probab* 2010;42:761–94.

[66] Hoops S, Sahle S, Gauges R et al. COPASI—a complex pathway simulator. *Bioinformatics* 2006;22:3067–74. 10.1093/bioinformatics/btl485 17032683

[67] Holme P, Saramäki J. Temporal networks. *Phys Rep* 2012;519:97–125.

[68] Perra N, Gonçalves B, Pastor-Satorras R et al. Activity driven modeling of time varying networks. *Sci Rep* 2012;2:469. 10.1038/srep00469 PMC3384079 22741058

[69] Cure S, Pflug FG, Pigolotti S. Fast and exact stochastic simulations of epidemics on static and temporal networks. *PLOS Comput Biol* 2025;21:e1013490. 10.1371/journal.pcbi.1013490 PMC12448994 40953134

[70] Pollard H . Representation of ${e}^{-{x}^{\lambda }}$ as a Laplace integral. *Bull Am Math Soc* 1946;52:908.

[71] Kanter M. Stable densities under change of scale and total variation inequalities. Ann Probab 1975;3:697–707.

[72] Deng Y, Wenjie D. The Kantorovich metric in computer science: a brief survey. In: *Electronic Notes in Theoretical Computer Science* 2009;253:73–82.

[73] Rubner Y, Tomasi C, Guibas LJ. A metric for distributions with applications to image databases. In: Sixth International Conference on Computer Vision (IEEE Cat. No. 98CH36271), Bombay, India: IEEE, pp. 59–66, 1998.

[74] Bonneel N, van de Panne M, Paris S et al. Displacement interpolation using Lagrangian mass transport. *ACM Trans Graph* 2011;30:1–12. 10.1145/2070781.2024192

[75] Pele O, Werman M. Fast and robust Earth Mover’s Distances. In: IEEE 12th International Conference on Computer Vision, Kyoto, Japan: IEEE, pp. 460–7, 2009.

[76] Davis K . A global optimization algorithm worth using. *dlib C++ library* 2017. http://blog.dlib.net/2017/12/a-global-optimization-algorithm-worth.html

[77] Snoek J, Larochelle H, Adams RP. Practical Bayesian optimization of machine learning algorithms. Adv Neural Inf Process Syst 2012;25:2951–9.

[78] Wand MP, Marron JS, Ruppert D. Transformations in density estimation. *J Am Stat Assoc* 1991;86:343–53.

[79] Herman J, Usher W. SALib: an open-source python library for sensitivity analysis. *J Open Source Softw* 2017;2:97.

