# Supplementary material for: Gillespie-based simulation and inference for non-Markovian stochastic reaction networks
Source: Brief Bioinform. 2026 Sep 22;27(5):bbag486. doi: 10.1093/bib/bbag486 (PMC13595353; doi:10.1093/bib/bbag486)
Supplement: Supplementary file 1 [file si_bbag486.pdf]

Supplementary Materials

Gillespie-Based Simulation and Inference for Non-Markovian Stochastic Reaction Networks

Aurelien Pelissier[1,2,3], **Miroslav Phan**[1,2], **Niko Beerenwinkel**[2], **Marıa Rodrıguez Martınez**[1,5,†]

[1]IBM Research Europe, 8803 Ruschlikon, Switzerland
[2]Department of Biosystems Science and Engineering, ETH Zurich, 4058 Basel, Switzerland
[3]Institute of Computational Life Sciences, Zurich University of Applied Sciences (ZHAW), 8820, Wadenswil, Switzerland
[4]Department of Biomedical Informatics & Data Science, Yale School of Medicine, New Haven, CT, United States
[†]Corresponding author maria.rodriguezmartinez@yale.edu.

A Non-Markovian division

A.1 Cell inter-division time

On Figure S1 and Table S1, we show the normal, Weibull, gamma and log-normal distributions with parameters chosen to fit experimentally measured cell cycle inter event times for *Bacillus subtilis* [1] and *Escherichia coli* [2] (`https://jun.ucsd.edu/repository.php`). The log-normal distribution provides the optimal fit in all three cases as it minimizes the Earth Mover's Distance (EMD) [3].

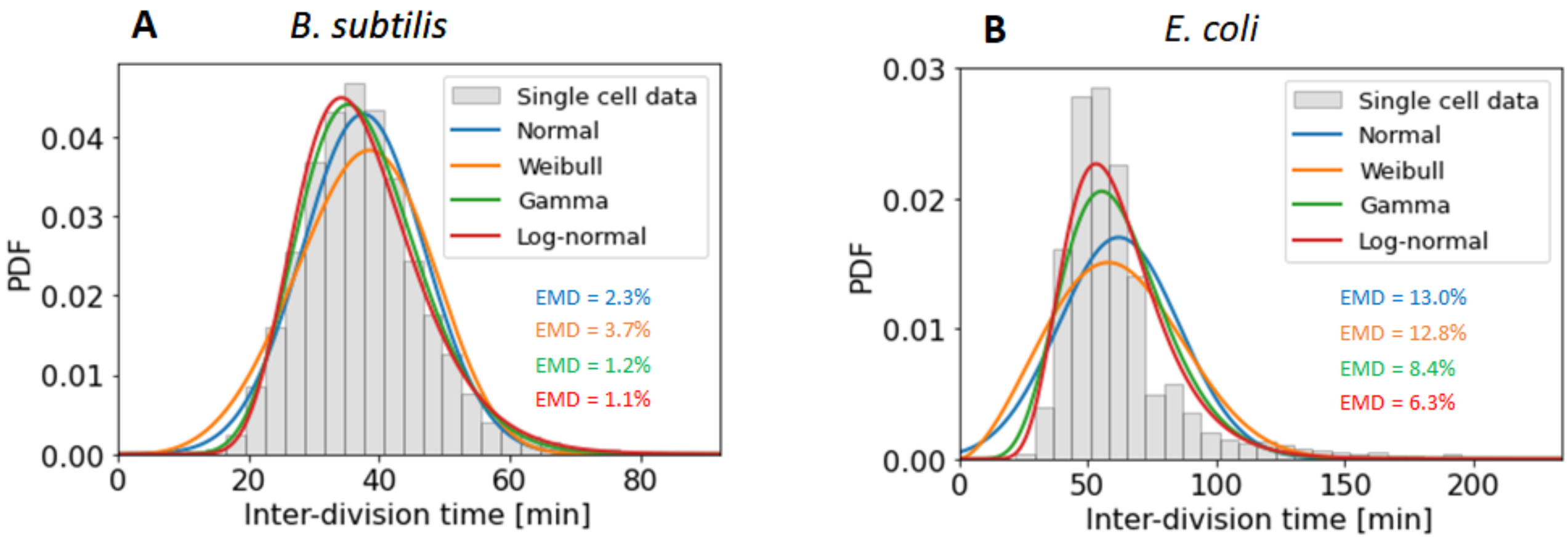

Figure S1: Measured interdivision time of (A) *Bacillus subtilis* [1] and (B) *Escherichia coli* [2] at constant temperature fitted with a normal, log-normal, gamma and Weibull distribution. The fitted distributions are quantified in term of the Earth Mover's Distance (EMD) to the measured single cell inter-division distribution.

Distribution	Exponential	Normal	Log-normal	Gamma	Weibull
EMD - *Bacillus subtilis* [1]	55.8	2.3	**1.1**	**1.2**	3.7
Optimal parameter ($1/\lambda_0$)	37.6 min	37.6 min	37.6 min	37.6 min	37.4 min
Optimal parameter (γ or α)	–	0.25	0.26	16.4	4.2
EMD - *Escherichia coli* [2]	47.4	13.0	**6.3**	8.4	12.8
Optimal parameter ($1/\lambda_0$)	62.0 min	62.0 min	61.7 min	62.0 min	61.8 min
Optimal parameter (γ or α)	–	0.38	0.32	9.3	2.6
EMD - *Caulobacter crescentus* [4]	64.9	2.1	**1.8**	**1.7**	5.0
Optimal parameter ($1/\lambda_0$)	72.1 min	72.1 min	72.1 min	72.1 min	71.4 min
Optimal parameter (γ or α)	–	0.12	0.12	70.8	7.1

Table S1: Fitting performance of various distributions to measured single cell inter-division time distribution of three types of bacteria dividing at constant temperature. The optimal parameters as well as the computed Earth Mover Distance (EMD) between the fitted and measured distributions are provided. EMDs are given with a confidence interval of $\pm 10\,\%$ of the provided value, due to stochaticity in the fitting process. Lowest EMD scores for each type of bacteria are highlighted in bold.

A.2 Symmetric division

A simple system following non-Markovian dynamics that can be described analytically is symmetric division, where a single type of reactant A reacts to produce $\nu > 1$ identical versions of itself. We thus consider the reaction $A \to \nu A$. In the general case, the population's steady state exponential growth rate k is related to the single-cell division time distribution $\psi(t)$ through the integral [5]

$$\mathcal{L}\{\psi\}(k) = \int_0^\infty e^{-k\tau}\psi(\tau)d\tau = \frac{1}{\nu}. \tag{1}$$

In words, k is the point at which the Laplace transform of the division time distribution $\psi(\tau)$ is equal to $1/\nu$. Then, the mean doubling time of the population is obtained with $t_2 = \ln(2)/k$. Defining the mean division time of single cells as $\mu = \int_0^\infty t\psi(t)dt$, we can solve the above equation for particular cases.

Deterministic processes. For a deterministic division (Dirac distribution), with PDF $\psi(t) = \delta(t-\mu)$, the Laplace transform is given by

$$\mathcal{L}\{\psi\}(k) = \exp(-\mu k). \tag{2}$$

Thus, we can simplify equation 1 into

$$\exp\left(-\frac{\ln(2)\cdot\mu}{t_2}\right) = \frac{1}{\nu} \quad \text{which yields} \quad \boxed{t_2 = \mu \cdot \frac{\ln(2)}{\ln(\nu)}.} \tag{3}$$

Markovian processes. For an exponential distribution, with PDF $\psi(t) = \lambda e^{-\lambda t}$, the Laplace transform is given by

$$\mathcal{L}\{\psi\}(k) = \frac{\lambda}{k+\lambda}. \tag{4}$$

Replacing $\lambda = 1/\mu$, we can simplify equation 1 into

$$\frac{1}{1+\mu\ln(2)/t_2} = \frac{1}{\nu} \quad \text{which yields} \quad \boxed{t_2 = \mu \cdot \frac{\ln(2)}{\nu - 1}.} \tag{5}$$

Non-Markovian processes. From Eq.1, the population doubling time can be computed exactly for any distributions with an analytical form of its Laplace transform. We provide an example with the gamma distribution, with PDF $\psi(t) = \frac{\beta^\alpha}{\Gamma(\alpha)}t^{\alpha-1}e^{-\beta t}$ and Laplace transform

$$\mathcal{L}\{\psi\}(k) = \frac{\beta^\alpha}{(\beta+k)^\alpha}. \tag{6}$$

Replacing $\beta = {}^{\alpha}/_{\mu}$, we can simplify equation 1 into

$$\frac{1}{1+\mu\ln(2)/(\alpha t_2)} = \left(\frac{1}{\nu}\right)^{1/\alpha} \quad \text{which yields} \quad \boxed{t_2 = \mu \cdot \frac{\ln(2)}{\alpha\left(\nu^{1/\alpha}-1\right)}} \qquad [7]$$

where $\alpha = 1$ recovers the Markovian case, and $\alpha = \infty$ the deterministic case[1]. We illustrate these result Figure S2, where the influence of the shape parameter on the doubling rate can be visualized. We simulate the non-Markovian division process with NoMaSS and fit the obtained population growth with an exponential (which result in a straight line when plotting with a logarithmic scale, Figure S2B). As expected from the analytical expression Eq.7, the obtained growth rate reduces with the shape parameter and, in the special case of $\nu = 2$, converge to $k = \ln 2$ for increasing α.

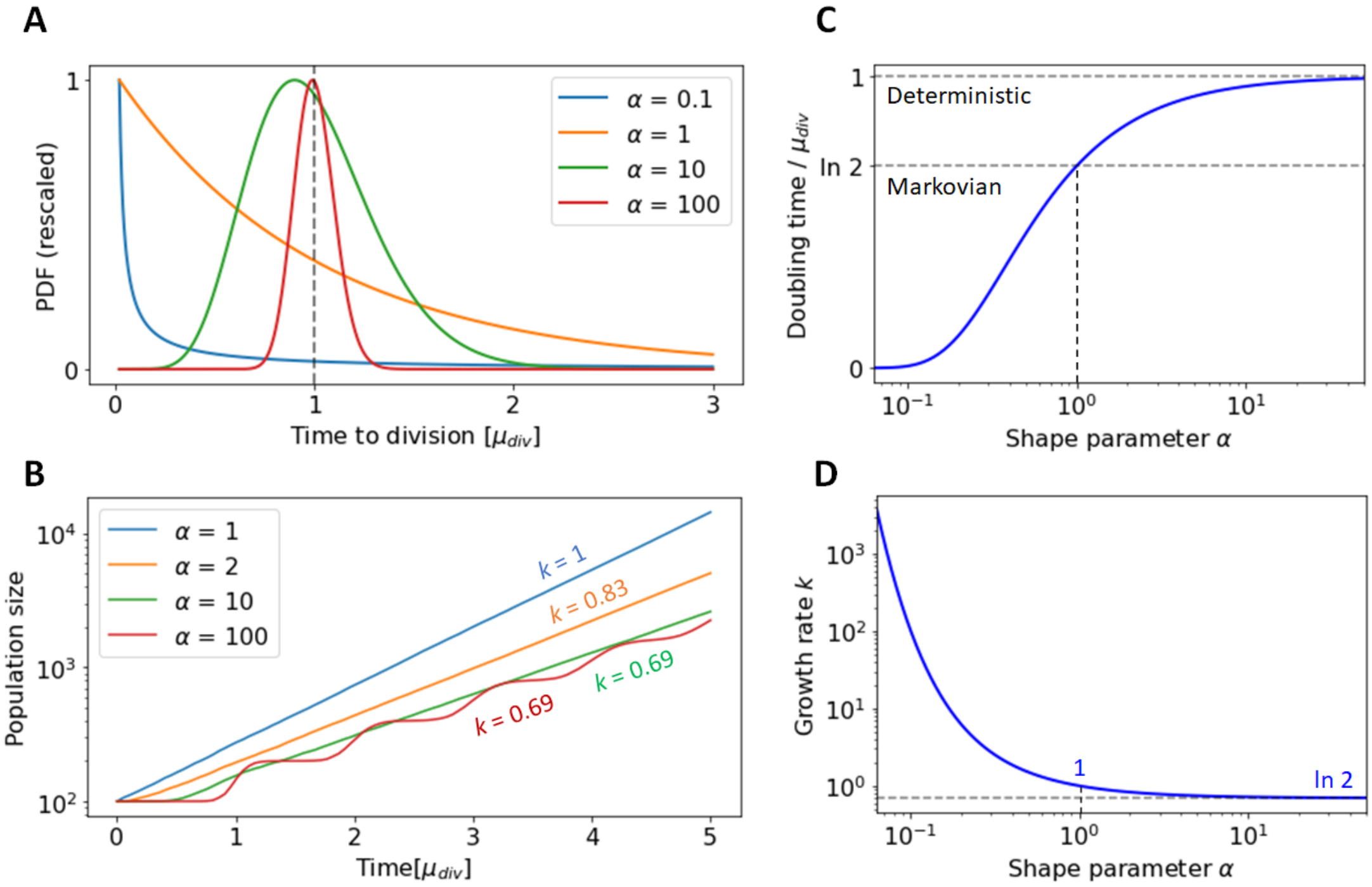

Figure S2: Population dynamics of Non-Markovian division following the equation $A \rightarrow 2A$ with a single cell interdivision time distribution following a gamma distribution of shape parameter α and mean μ_{div}. (A) PDF of gamma distributions with mean 1 and shape parameter α, rescaled for visual clarity. (B) Population growth of A for different shape parameter simulated with NoMaSS. The fitted exponential growth rate (k) is provided above each line. (C) Analytical ratio between the population doubling time ($t_2 = \ln(2)/k$) and the single cell average division time for different value of α. (D) Analytical growth rate of the population size for different value of α. Special cases for the Markovian ($\alpha = 1$) and deterministic ($\alpha \rightarrow \infty$) divisions are annotated.

A.3 Asymmetric division

A more complex non-Markovian system, that we describe in the main text, is asymmetric division, where a reactant A reacts to produce ν_A versions of itself as well as ν_B versions of another reactant B, following the reaction (i) $A \rightarrow \nu_A A + \nu_B B$. Then, reactant B does not divide but can differentiate into A following the reaction (ii) $B \rightarrow A$. In this setup, it was shown analytically that the population of both reactants A and B grow exponentially with the same rate k for any inter-event distribution of reactions (i) and (ii), where the growth rate k is related to the parameters ν_A and ν_B with the relation [5]

$$\mathscr{L}\{\psi_{\text{div}}\}(k) = \frac{1}{\nu_A + \nu_B \cdot \mathscr{L}\{\psi_{\text{diff}}\}(k)}, \qquad [8]$$

[1]Applying l'Hôpital's rule, It can be shown that $\lim_{x\to\infty}(xa^{1/x} - x) = \ln(a)$

where ψ_{div} and ψ_{diff} denote the PDF of reaction (i) and (ii), respectively. In the Markovian case, one can solve this equation with:

$$\frac{\lambda_{\text{div}}}{k+\lambda_{\text{div}}} = \frac{k+\lambda_{\text{diff}}}{\nu_A(k+\lambda_{\text{diff}})+\nu_B\lambda_{\text{diff}}}, \quad [9]$$

which yields the quadratic equation

$$k^2 + k\cdot(\lambda_{\text{div}} - \nu_A\lambda_{\text{div}} + \lambda_{\text{diff}}) + \lambda_{\text{div}}\lambda_{\text{diff}}(1-\nu_A-\nu_B) = 0, \quad [10]$$

where we take the positive solution for the growth rate

$$k = \frac{1}{2}\left(\lambda_{\text{div}}\nu_A - \lambda_{\text{div}} - \lambda_{\text{diff}} + \sqrt{(\lambda_{\text{diff}}+\lambda_{\text{div}}-\lambda_{\text{div}}\nu_A)^2 + 4\lambda_{\text{div}}\lambda_{\text{diff}}(\nu_A+\nu_B-1)}\right). \quad [11]$$

Unconstrained microbial growth of *Caulobacter crescentus*. We now consider the specific setting of asymmetric division with $\nu_A = \nu_B = 1$ (Figure S4A), corresponding to the unconstrained microbial growth of *Caulobacter crescentus*. In the Markovian case, we can write

$$k = \frac{\lambda_{\text{diff}}}{2}\left(\sqrt{1+4\frac{\lambda_{\text{div}}}{\lambda_{\text{diff}}}} - 1\right). \quad [12]$$

In the limit $\lambda_{\text{div}} \ll \lambda_{\text{diff}}$, using the Taylor expansion $\sqrt{1+x} \sim 1 + x/2$, we obtain $k = \lambda_{\text{div}}$, which recovers the symmetric case ($A \to 2A$). In this regime, differentiation ($A \to B$) is very fast compared to division, so that it can be considered to occur instantly. On the other hand, in the limit where differentiation is very slow compared to division ($\lambda_{\text{div}} \gg \lambda_{\text{diff}}$), the growth rate is given by $k = \sqrt{\lambda_{\text{div}}\lambda_{\text{diff}}}$. Finally, in the specific case of $\lambda_{\text{div}} = \lambda_{\text{diff}} = \lambda$, we obtain $k = \lambda/\varphi$ where $\varphi = \frac{1+\sqrt{5}}{2}$ is the golden ratio.

In the general non-Markovian case (i.e. gamma or Dirac IEDs), there is no simple analytical solutions for k. Thus, we use NoMaSS to show how the growth rate varies (Figure S3) for different combinations of distribution parameters $(\lambda_{\text{div}}, \alpha_{\text{div}})$ and $(\lambda_{\text{diff}}, \alpha_{\text{diff}})$. As expected, changes in the mean of the distribution have a much larger

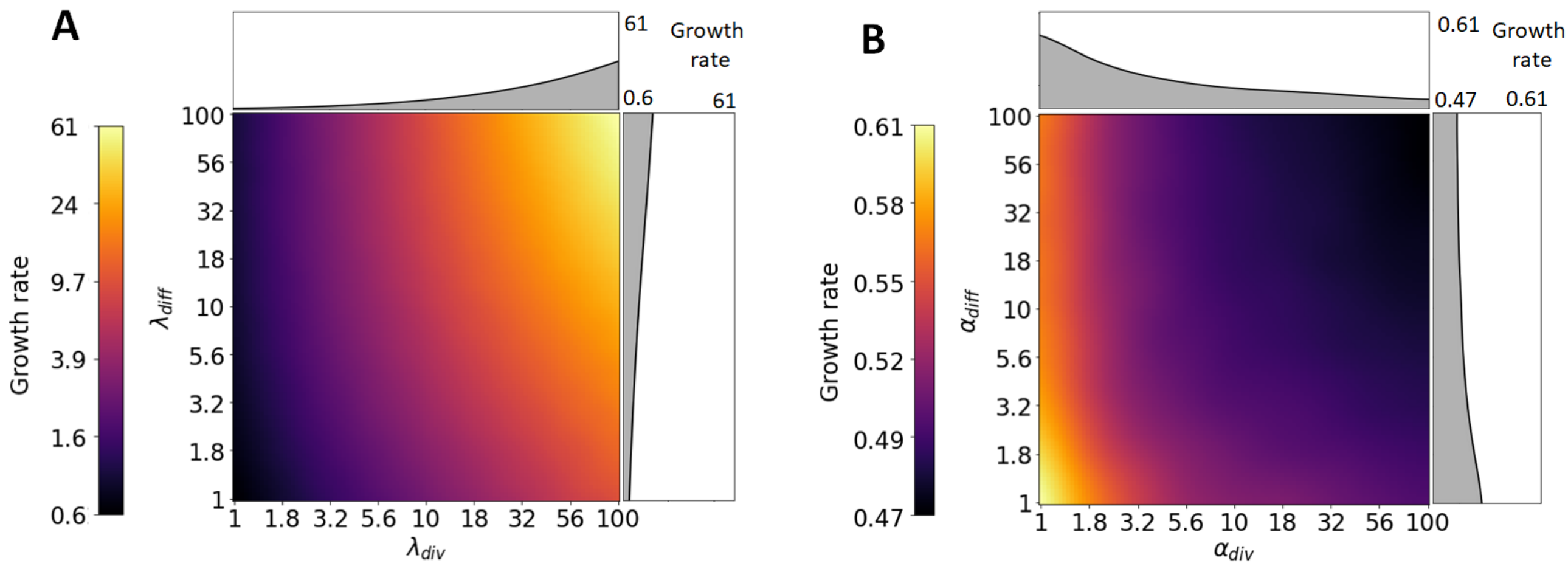

Figure S3: Growth rate of the number of cells ($N_A + N_B$) in a system of asymmetric division ($A \to A + B$ and $B \to A$), where the two processes are modeled as a gamma distribution with shape parameter $(\lambda_{\text{div}}, \alpha_{\text{div}})$ and $(\lambda_{\text{diff}}, \alpha_{\text{diff}})$. (A) Heatmap of the growth rate as a function of the processes rate $(\lambda_{\text{div}}, \lambda_{\text{diff}})$ where $\alpha_{\text{div}} = \alpha_{\text{diff}} = 1$ are kept constant. Note that growth rate axes are in logarithmic scales (B) Heatmap of the growth rate as a function of the processes shape parameters $(\alpha_{\text{div}}, \alpha_{\text{diff}})$ where $\lambda_{\text{diff}} = \lambda_{\text{div}} = 1$ are kept constant. The projection of the growth rate averaged over the differentiation and division parameter axis are plotted on the right and the top of the heatmap, respectively.

impact on the growth rate than changes in the shape parameter. Nevertheless, the shape parameter can affect the growth rate by more than ∼20%, highlighting the importance of properly accounting for it. Interestingly, we observe that α_{div} and α_{diff} affects the growth rate in a similar manner.

Oscillation amplitudes of quiescent cells. After fitting the model parameters with experimental data from [4], consisting of single-cell measurements of division times, as well as steady-state cell populations ratios of stalked and quiescent cells at 31 °C, we were able to constrain 3 out of 4 model parameters (Figure S4B,C).

However, we were not able to constrain α_{diff} as the data only provides information about steady-state levels for quiescent cell differentiation – for technical reasons, the direct experimental characterization of quiescent cell differentiation is challenging [6]. As previously shown [5], the deterministic modeling of quiescent cell differentiation reveals transient oscillations, as the initial populations are highly synchronized and differentiate at the same time. Stochastic models should partially smooth these transient oscillations, as division and differentiation occur probabilistically, which results in the gradual loss of synchronization. However, we see that in the Markovian case the oscillations have completely disappeared (Figure S4D), i.e. the exponentially distributed inter-event times fully mix division and differentiation times destroying the synchronization. Opposed to this, the non-Markovian simulation still shows transient oscillations (Figure S4E). Interestingly, the amplitude of the quiescent cell population oscillations increases as the variance of the inter-event differentiation time of stalked cells reduces (Figure S4F). We note that with our parametrization, α_{diff} inversely correlate to the variance of the distribution through the relation $Var = 1/(\alpha_{\text{diff}}\lambda_{\text{diff}}^2)$.

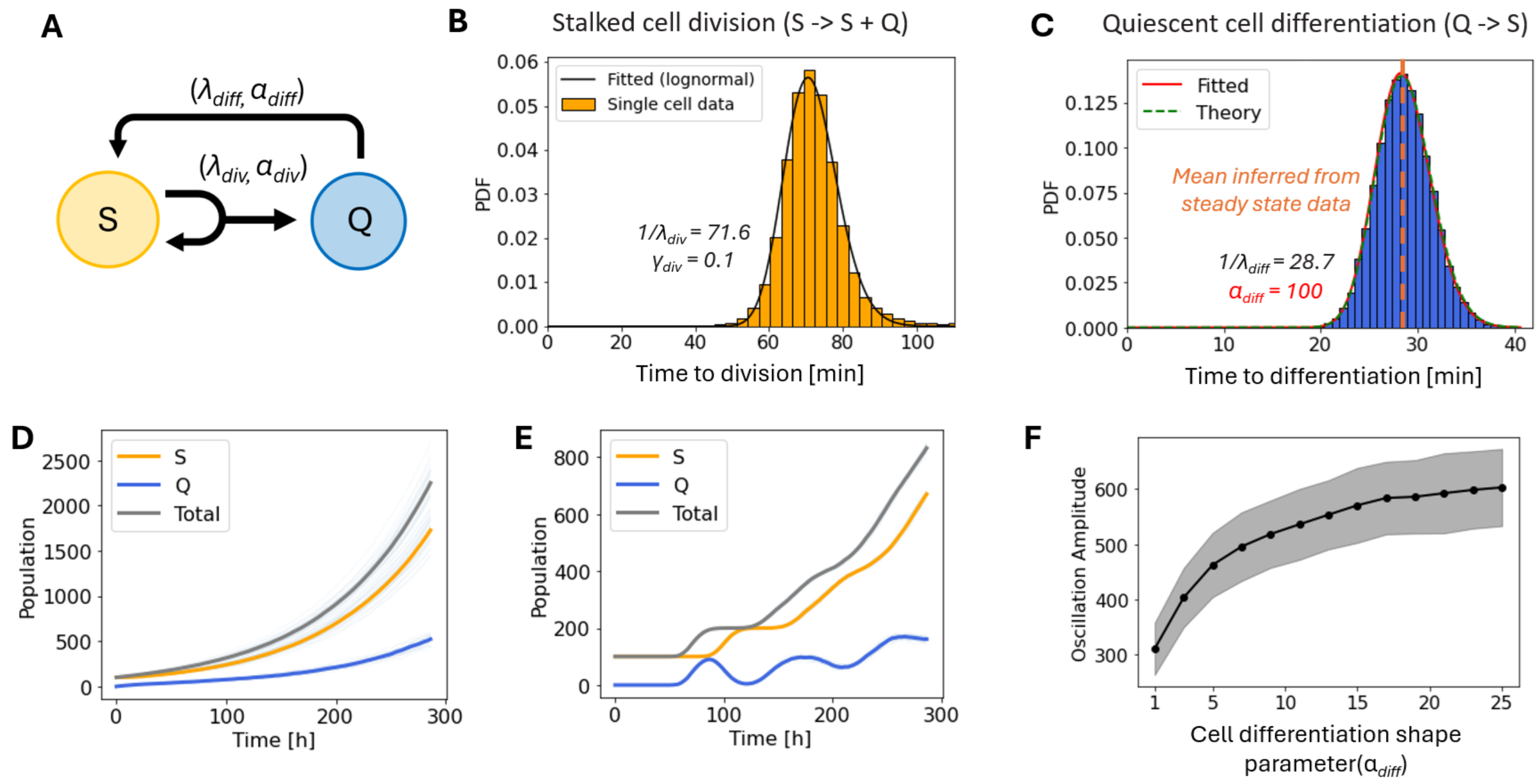

Figure S4: Microbial growth of *Caulobacter crescentus*. (A) Cartoon depicting the dynamics of microbial growth. A stalked cell S divides asymmetrically into another stalked cell and a motile quiescent cell Q that cannot divide further but can differentiate into a stalked cell. (B) Inter-event time distribution of stalked cell divisions using single-cell measurements from [4]. Data has been fitted to a log-normal distribution. The fitted distribution was later used in the NoMaSS simulation. (C) Simulated inter-event time of quiescent cell differentiation with a gamma distribution of shape parameter $\alpha_{\text{diff}} = 100$, chosen arbitrarily for the purpose of illustration (as there is no experimental data available). (D,E) Simulated population dynamics with an initial population of 100 stalked cells using Markovian processes and non-Markovian dynamics, respectively, both with the same mean inter-event time, and with the inter-event distributions taken from subplots B & C for the non-Markovian case. (F) Evolution of the oscillation amplitudes as a function of α_{diff} while keeping all other parameters constant (as shown on subplots B & C). The black line shows the mean of 1000 simulations, and the shaded area corresponds to one standard deviation. The oscillations are quantified in terms of the root mean square deviation (RMSD) between the quiescent cells population dynamics and its exponential fit.

B Modeling stem cell differentiation

This section refers to the modeling of mouse stem cell differentiation [7] dynamics. In this experiment, cells are profiled at the single-cell level at 3 different stages of differentiation (denoted A,B,C), thus undergoing two transitions ($A \to B$ and $B \to C$). For consistency across timepoints, measured population of N_A, N_B and N_C are transformed in term of proportion $(p_A, p_B, p_C) \in [0,1]^3$ following

$$p_A = \frac{N_A}{N_A + N_B + N_C}, \quad p_B = \frac{N_B}{N_A + N_B + N_C} \quad \text{and} \quad p_C = \frac{N_C}{N_A + N_B + N_C}. \qquad [13]$$

As the population dynamics is described by proportions bounded between 0 and 1. This may bias the the optimization of the root mean square deviation (RMSD) towards ignoring variations close to very low (~ 0) and very high (~ 1) values, as a proportion of 98 % will be considered closer to 99 % than 50 % is to 52 %. To correct for this bias, we transform both simulated and measured populations with an inverse sigmoid (or logit) function (Figure S5). For experimental reasons, proportions lower than 1% or higher than 99% are considered not reliable enough (only a few hundred cells in experimental data), so we only apply the transformation within that range:

$$\begin{aligned} y_{\text{new}} &= \log\left(\frac{y}{1-y}\right) \quad \text{for} \ \ 1\,\% \leq y \leq 99\,\% \\ y_{\text{new}} &= 4.6 \ \ \text{for} \ \ y > 99\,\% \\ y_{\text{new}} &= -4.6 \ \ \text{for} \ \ y < 1\,\% \end{aligned} \qquad [14]$$

With NoMaSS, we model the two differentiations processes with different choices of inter-event time distribution, and we provide in Table S2 the distribution parameters of the optimal fit to both the raw (unstransformed) and logit transformed experimental data. Optimal parameters were obtained by minimizing the RMSD between the measured and simulated populations at each time point. Minimization was performed with the `maxLIPO` algorithm [8], which is both parameter free and provably better than a random search. Unsurprisingly, the exponential distribution consistently yields to the highest RMSD, while the normal, log-normal and gamma output similarly low RMSD. On the other hand, the Weibull distribution consistently output higher RMSD than other distributions for both the untransformed and transformed data.

Distribution	Exponential	Normal	Log-normal	Gamma	Weibull
RMSD (sigmoid transformed data)	2.1	0.38	0.39	**0.35**	0.69
Optimal parameter ($1/\lambda_1$)	29.4 h	31.9 h	32.3 h	31.9 h	32.2 h
Optimal parameter ($1/\lambda_2$)	66.7 h	66.7 h	62.9 h	65.4 h	63.3 h
Optimal parameter (γ_1 or α_1)	–	0.24	0.21	20.2	4.3
Optimal parameter (γ_2 or α_2)	–	0.15	0.15	38.7	4.8
RMSD (untransformed data)	0.22	0.017	**0.014**	0.019	0.039
Optimal parameter ($1/\lambda_1$)	31.9 h	32.1 h	31.6 h	29.5 h	31.8 h
Optimal parameter ($1/\lambda_2$)	63.7 h	59.2 h	59.9 h	61.0 h	58.8 h
Optimal parameter (γ_1 or α_1)	–	0.27	0.27	27.6	4.65
Optimal parameter (γ_2 or α_2)	–	0.050	0.083	39.3	4.94

Table S2: Root mean square deviation (RMSD) between simulated and measured stem cell differentiation data for both the raw and sigmoid transformed data. Lowest RMSD scores are highlighted in bold.

C Non-Markovian dynamics of RNA transcription

This section provide additional information regarding the two steps model of RNA transcription described in the main text. As a reminder, we consider a promoter switching between an ON and an OFF state with gamma inter-event distributions parametrized by $(\lambda_{\text{on}}, \alpha_{\text{on}})$ and $(\lambda_{\text{off}}, \alpha_{\text{off}})$. Some mRNA may be produced only if the promoter state is ON, with a gamma inter-event distribution $(\lambda_{\text{prod}}, \alpha_{\text{prod}})$. The mRNA then degrades at a constant rate λ_{deg}. The system is initialized with a promoter in an OFF state and a count of 0 mRNA.

C.1 Oscillations of mRNA dynamics

Modeling RNA transcription with non-Markovian processes may results into predictable oscillations of averaged mRNA counts before the steady state is reached, a phenomenon that is not observed in the Markovian case.

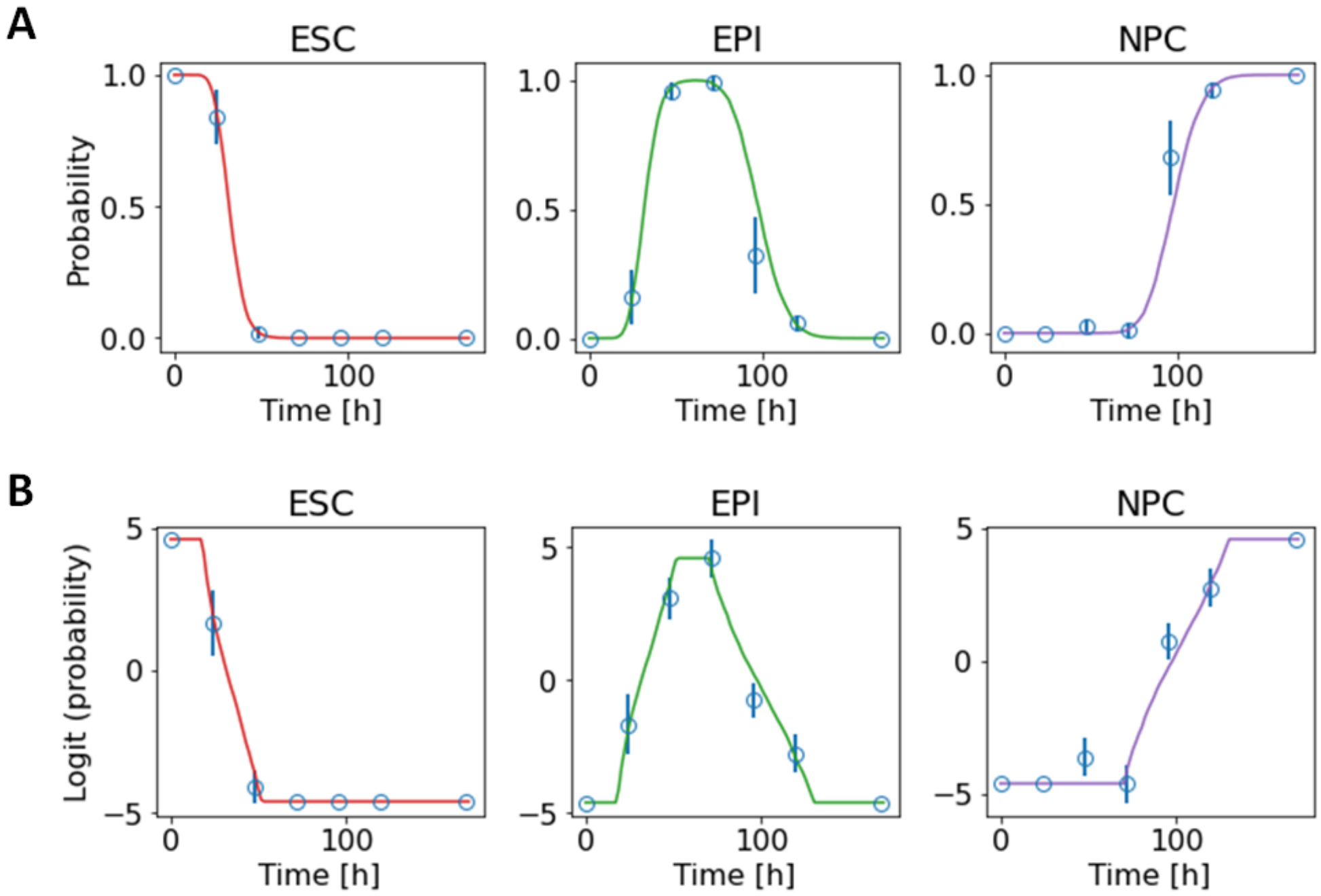

Figure S5: Normalized stem cell population as a function of time for the three stages of differentiations following the reactions ESC → EPI and EPI → NPC. The blue points represent the mean and standard deviation of the stem cell populations measured at different time points [7], and the solid lines depict the population dynamics averaged over 1000 NoMaSS simulation, with differentiation modelled as the best fitting gamma distribution. Results are shown for (A) the untransformed and (B) transformed probabilities with an inverse sigmoid (or logit) function.

For example, in the specific case of a deterministic promoter transition (promoter activation and deactivation modeled as Dirac distributions), the promoter oscillates between the G_{on} and G_{off} state indefinitely with a period of $T = 1/\lambda_{on} + 1/\lambda_{off}$, which results in oscillations in mRNA count with the same period. Adding some stochasticity to the promoter transition results in a decay of the G_{on} and G_{off} probability oscillation amplitude, until it converges to the steady state value and no more amplitudes can be observed (Figure S6A&B). Interestingly, the more *stochasticity* is added to the promoter transition reaction (decreasing shape parameter α_{on} and α_{off}), the faster the oscillation decays (Figure S6B&C). In the case of Markovian mRNA transcription ($\alpha_{on} = \alpha_{off} = 1$), the system reaches directly its steady state after a single period T and oscillations are not observed (Figure S6A).

C.2 Modeling the reaction to stimuli in RNA transcription

Non steady state RNA transcription dynamics are not straight forward to observe directly experimentally. A possibility is to study the behavior of RNA dynamics after stimuli by the epidermal growth factor (EGF) or lipopolysaccharide (LPS), which initiate well characterized signaling cascades that culminate in orchestrated transcriptional responses [9]. After the stimuli, the system take some times to reach its new steady state, which results in observable non-steady state dynamics shortly after the stimuli. It was shown that the stimuli may affect all rates involved in RNA transcription ($\lambda_{on}, \lambda_{off}, \lambda_{deg}$) [9] (Figure S7B). To avoid introducing too many new parameters in our model to incorporate the stimuli, we keep λ_{deg} and λ_{off} constant, and we set the promoter activation rate $\lambda_{on}(t)$ to change with time as a function of two terms:

$$\lambda_{on}(t) = \lambda_{on}^{decay}(t) + \lambda_{on}^{peak}(t)\,. \qquad [15]$$

The first one is a progressive decay (modeled with a sigmoid function) from its value before the stimuli (λ_A) to its new value (λ_B) as

$$\lambda_{on}^{decay}(t) = (\lambda_A - \lambda_B) \cdot \frac{e^{-b\,(t-t_0)}}{1 + e^{-b\,(t-t_0)}} + \lambda_B \qquad [16]$$

where t refers to the time elapsed since the stimuli. Additionally, we also add a second term to simulate the short term peak induced by the stimuli, as introduced in original study [9]:

$$\lambda_{on}^{peak}(t) = a_1 \frac{(t/t_p)^{n_1}}{(1 + t/t_p)^{n_2}}. \quad \text{with} \quad n_1 < n_2 \qquad [17]$$

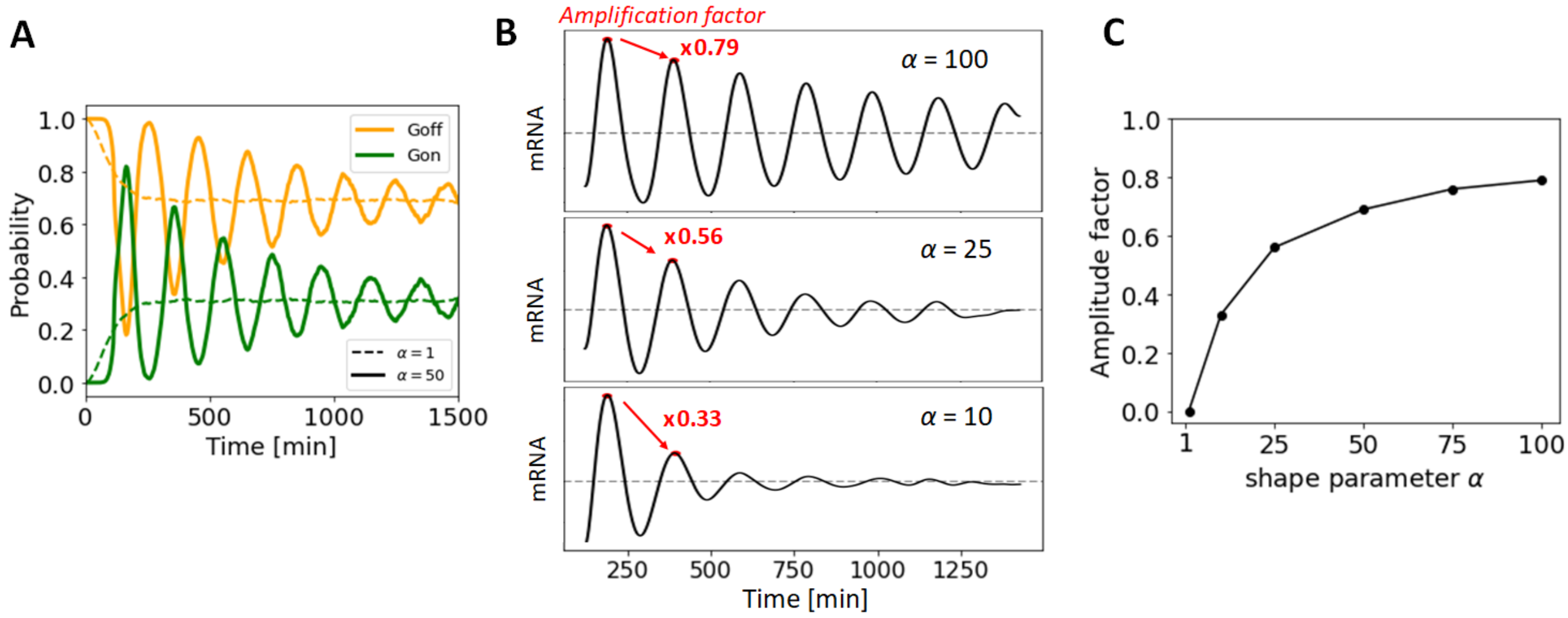

Figure S6: Oscillations in non-Markovian mRNA transcription dynamics where we set $\alpha = \alpha_{\text{on}} = \alpha_{\text{off}}$ for simplicity. (A) Time dependent probability that the promoter is in the on or off states for both the Markovian ($\alpha = 1$, dashed line) and non Markovian case ($\alpha = 50$, solid line) at each time point, obtained after averaging over 10k simulations. (B) Time evolution of mRNA count averaged over 10k NoMaSS simulations for different shape parameters α. For visual clarity, only the time interval where the mRNA count oscillate around its steady state value (dashed line) is plotted. The observed factor at which the oscillation amplitude decays between each period is shown is red. (C) Dependency of the shape parameter α on the oscillation amplification factor (amplitude of the second peak divided by the amplitude of the first peak) of averaged mRNA counts.

This peak induce a very short spike in the λ_{on} rate (Figure S7B) and cause the system to leave its steady state to enter non-Markovian dynamics. To make the parameter optimization easier, we fix the parameters $t_p = 5\,\text{min}$, $n_1 = 2$ and $n_2 = 4$ for all our simulation. On the other hand, we leave the parameter a_1 as variable since it reflects the amplitude of the peak and thus the oscillation amplitudes of RNA transcription.

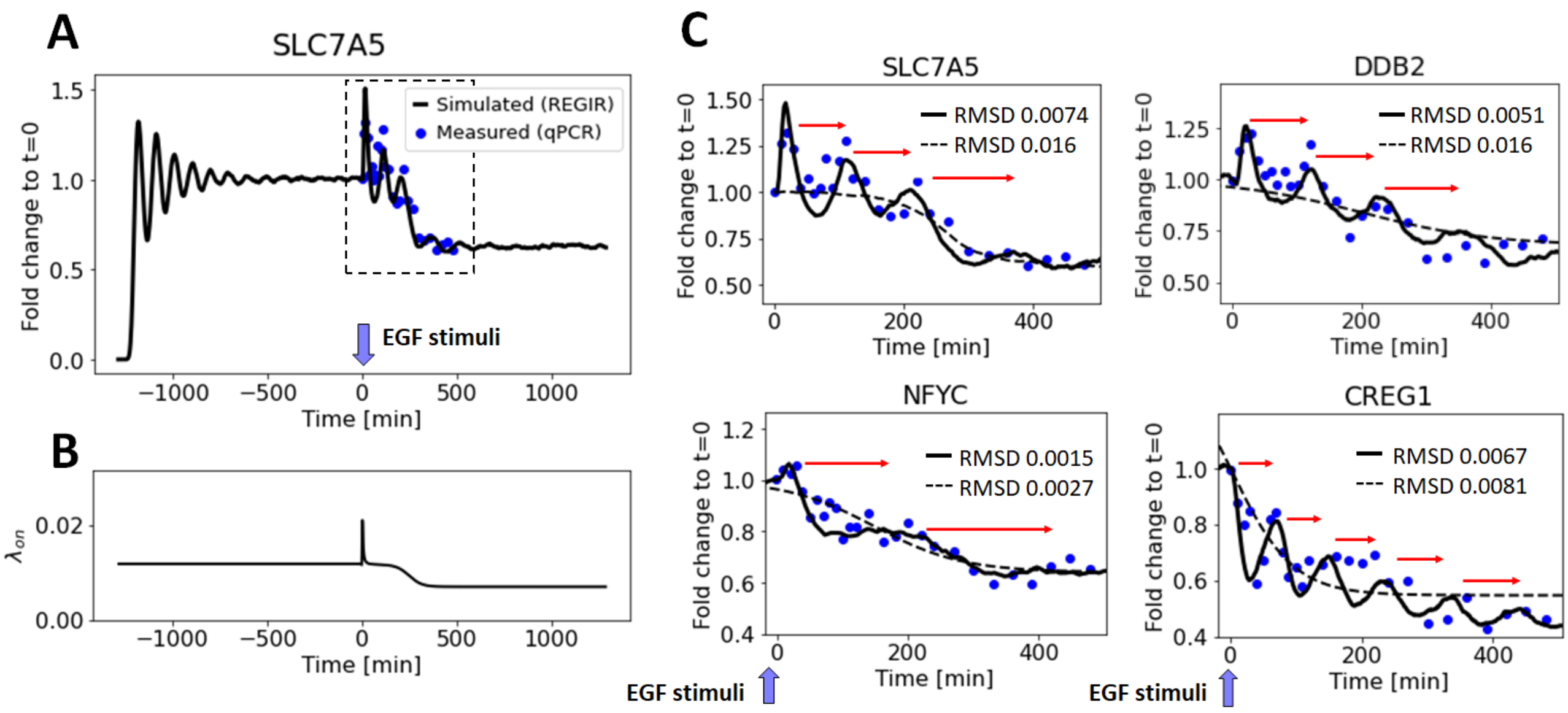

Figure S7: (A) Simulated mRNA gene expression of SLC7A5 normalized by its average expression at the time of stimulation, averaged over 10k simulation. We can clearly distinguish two phases, where the mRNA dynamics reaches first reaches its steady state and then reacts to the stimuli. (B) Time evolution of the simulated promoter activation rate of SLC7A5 (λ_{on}) before ($t < 0$) and after ($t > 0$) the EGF stimuli. (C) mRNA gene expression of different genes normalized by their average expression at the time of stimulation, where the measured expression [9] by quantitative PCR (qPCR) are compared to simulated RNA expression averaged over 10k simulations with optimal parameter fit. The simulation is provided for both NoMaSS (solid line) and the Markovian model (SG, dashed line). Red arrow highlight the increase in oscillation period caused by the decrease of λ_{on}.

The modeling of stimuli is performed by first simulating the system before the stimuli until its steady state is reached ($\lambda_{\text{on}} = \lambda_A$), and then re-initializing $t = 0$ and the promoter to its off state (Figure S7A). We adjust the models parameters to optimize the fit to experimental data for the genes CREG1, DDB2, SLC7A5, and NFYC (Figure S7C). For all simulation, we constrain $\alpha_{\textbf{on}} = \alpha_{\textbf{off}} = \alpha$ and $\alpha_{\textbf{prod}} = 1$ to facilitate the parameter fit. Note that with these constraints, the non-Markovian model only have one more parameter of freedom (α) than the Markovian model. For each gene, parameters (α, λ_A, λ_B, b, t_0 and a_1) are fitted with the `maxLIPO` algorithm [8] by minimizing the RMSD between the NoMaSS output of 1000 simulations and the experimental data (Table S3). Note that since the dataset involve fold-change of mRNA since $t = 0$ rather than absolute mRNA count, it is not possible to fit parameters such as λ_{off} or λ_{prod}, so we set them to constant values for all simulations ($\lambda_{\text{off}} = 4\lambda_{\text{deg}}$ and $\lambda_{\text{prod}} = 25\lambda_{\text{deg}}$). Also, while $\lambda_{\text{on}}(t = 0) = \lambda_A$ can be inferred from the oscillation period in the non-Markovian model (NoMaSS), it is not possible to obtain it in the Markovian case (SG) as there is no oscillations (marked as X in Table S3).

Interestingly, several of the studied genes revealed oscillations in their mRNA expression after the stimuli that could not be replicated accurately by the Markovian model (e.g. RMSD = 0.016 for SLC7A5). On the other hand, NoMaSS was able to reproduce the oscillations accurately (e.g. RMSD = 0.0083 for SLC7A5). Strikingly, the genes where the most significant difference was observed between the non-Markovian and Markovian model were the one with the highest oscillation amplitudes. Another interesting aspect is the gradual increasing period of the oscillations, which indicate a progressive reduction in the promoter activation rate (red arrows on Figure S7C).

	RMSD	λ_A	λ_B	b [1/min]	t_0 [min]	a_1	α
CREG1 (SG)	0.0081	X	$0.45 \cdot \lambda_A$	0.004	0	X	$\vert$
CREG1 (NoMaSS)	**0.0067**	$\lambda_{\text{off}}/4.5$	$0.45 \cdot \lambda_A$	0.006	0	-1.2	40
DDB2 (SG)	0.016	X	$0.66 \cdot \lambda_A$	0.029	214	X	$\vert$
DDB2 (NoMaSS)	**0.0051**	$\lambda_{\text{off}}/9$	$0.66 \cdot \lambda_A$	0.012	250	1	60
NFYC (SG)	0.0027	X	$0.64 \cdot \lambda_A$	0.015	150	X	$\vert$
NFYC (NoMaSS)	**0.0015**	$\lambda_{\text{off}}/18$	$0.64 \cdot \lambda_A$	0.008	160	0.55	15
SLC7A (SG)	0.016	X	$0.63 \cdot \lambda_A$	0.041	270	X	$\vert$
SLC7A (NoMaSS)	**0.0074**	$\lambda_{\text{off}}/9.6$	$0.63 \cdot \lambda_A$	0.035	270	1.3	26

Table S3: Optimal parameters and root mean square deviation (RMSD) between the simulated and measured mRNA time resolved gene expression fold changes after EGF stimulation [9]. Results are provided for both the SG and NoMaSS model, each averaged over 1000 simulations. Best RMSD scores for each genes are highlighted in bold. Parameters marked with | indicate that it is not applicable in the model. Parameters marked with X mean that the model is insensitive to its value, so it's irrelevant in the context of optimization.

C.3 Phase diagrams and sensitivity analysis of the steady state mRNA count distribution

In the main text, we performed a variance-based sensitivity analysis of the coefficient of variation (CV = std/mean) of the steady state mRNA distribution. In Figure S8, we provide the same sensitivity analysis for the mean, and the differential entropy H. Intuitively, H measures the randomness of a random variable and the number of bits required to describe it. Formally, H is computed as a generalization of the Shannon entropy to continuous

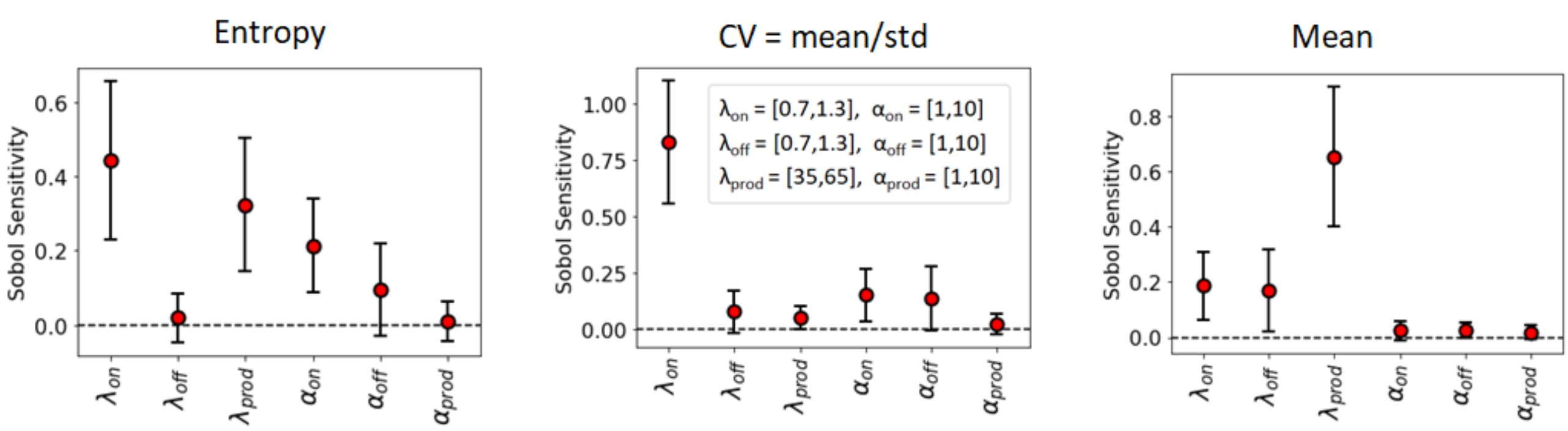

Figure S8: Sobol variance-based sensitivity analysis of the Mean, CV and entropy of the steady state single cell mRNA distribution generated by the two step model, with a grid of 128 points per parameters. The parameter bounds used for all analysis are given in the middle box.

distributions:

$$H(X) = -\int_{\mathcal{X}} f(x)\ \log f(x)\ dx. \tag{18}$$

Numerically, we compute it by discretizing the mRNA distribution into N_b bins and computing the histogram. As the choice of the number of bin can affect the measure, we chose the same number of bins $N_b = 25$ for all entropy estimations.

The sensitivity analysis of the mean steady state mRNA distribution is consistent with previous analytical results showing that, in the steady state, the mean mRNA distribution only depends on the mean inter-event time distribution [10], and thus, is not influenced by the shape parameters. We also compute phase diagrams

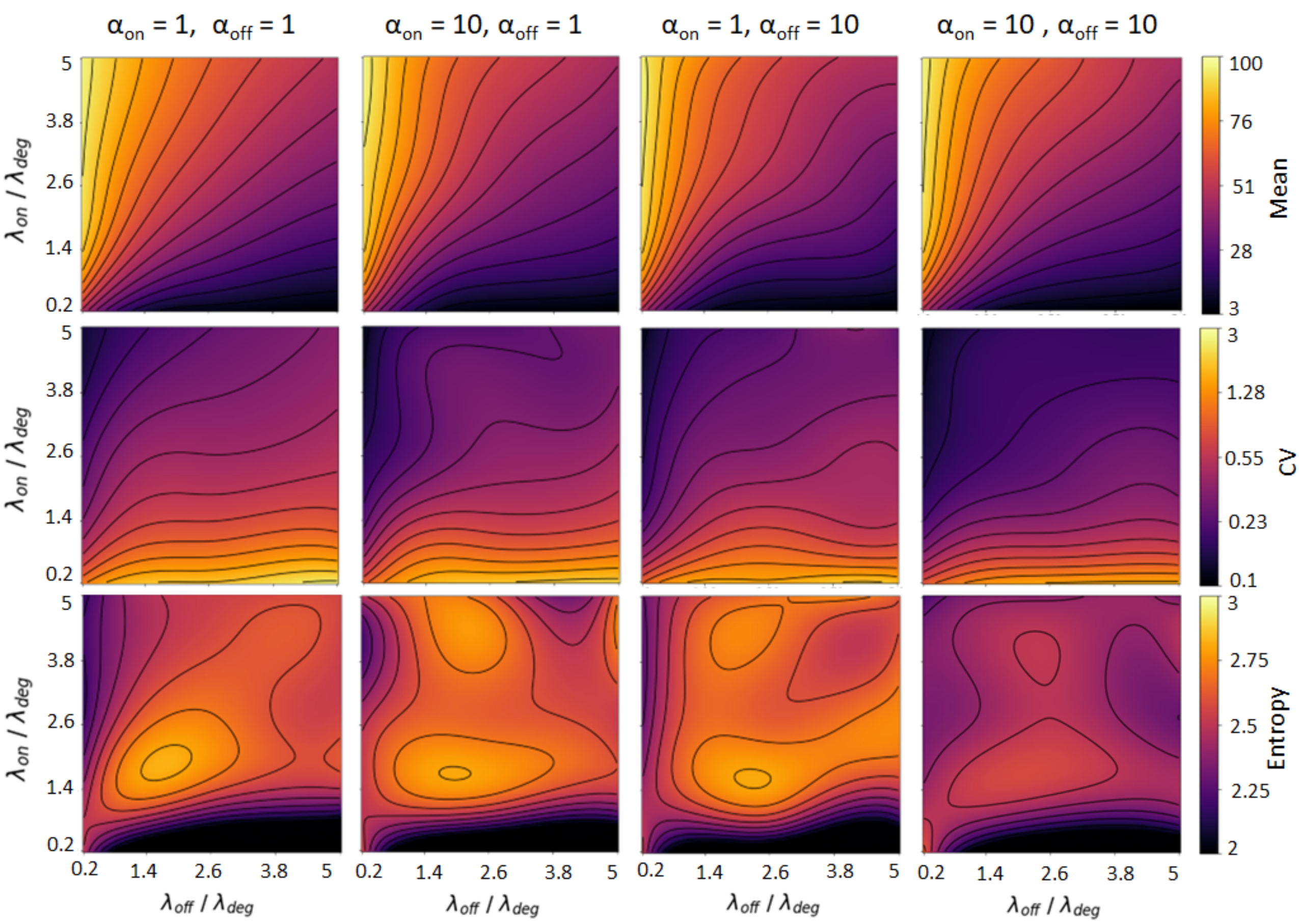

Figure S9: Mean, CV and entropy phase diagram of the steady state single cell mRNA distribution for different combination of shape parameter, i.e. the Markovian ($\alpha_{\text{on}} = \alpha_{\text{off}} = 1$) and non-Markovian cases ($\alpha_{\text{on}} \neq 1, \alpha_{\text{off}} \neq 1$). The heat map were generated with a 2D cubic interpolation of a 10x10 parameter grid, with NoMaSS results averaged over 1000 simulations. Parameters $\alpha_{\text{prod}} = 1$ and $\lambda_{\text{prod}} = 100$ were fixed for all simulations.

of the mean, CV and entropy of mRNA distribution, where the impact of variation in the parameters can be assessed visually. On Figure S9, we show it for the combinations ($\alpha_{\text{on}} = 1, \alpha_{\text{off}} = 1$), ($\alpha_{\text{on}} = 10, \alpha_{\text{off}} = 1$), ($\alpha_{\text{on}} = 1, \alpha_{\text{off}} = 10$) and ($\alpha_{\text{on}} = 10, \alpha_{\text{off}} = 10$). We observe that, although sensitive, changing the shape parameters (α_{on}, α_{on}) do not affect significantly the phase diagrams of the CV, but rather lower all CV values by a constant factor (0.4 in this case). As for the entropy, the variations across the depicted parameter space is relatively small except for a low λ_{on} where it shows a drastic decrease. We note that the contour plot in the phase diagrams are relatively noisy due to the low variation of the entropy in the simulated parameter range.

Bibliography

[1] John T Sauls et al. “Control of Bacillus subtilis replication initiation during physiological transitions and perturbations”. In: *MBio* 10.6 (2019), e02205–19.

[2] Fangwei Si et al. “Mechanistic origin of cell-size control and homeostasis in bacteria”. In: *Current Biology* 29.11 (2019), pp. 1760–1770.

[3] Yossi Rubner, Carlo Tomasi, and Leonidas J Guibas. "A metric for distributions with applications to image databases". In: *Sixth International Conference on Computer Vision (IEEE Cat. No. 98CH36271)*. IEEE. 1998, pp. 59–66.

[4] Srividya Iyer-Biswas et al. "Scaling laws governing stochastic growth and division of single bacterial cells". In: *Proceedings of the National Academy of Sciences* 111.45 (2014), pp. 15912–15917.

[5] Farshid Jafarpour et al. "Bridging the timescales of single-cell and population dynamics". In: *Physical Review X* 8.2 (2018), p. 021007.

[6] Jennifer C England et al. "Global regulation of gene expression and cell differentiation in Caulobacter crescentus in response to nutrient availability". In: *Journal of bacteriology* 192.3 (2010), pp. 819–833.

[7] Patrick S Stumpf et al. "Stem cell differentiation as a non-Markov stochastic process". In: *Cell Systems* 5.3 (2017), pp. 268–282.

[8] King Davis. "A Global Optimization Algorithm Worth Using". In: *dlib C++ library*. 2017. URL: http://blog.dlib.net/2017/12/a-global-optimization-algorithm-worth.html.

[9] Amit Zeisel et al. "Coupled pre-mRNA and mRNA dynamics unveil operational strategies underlying transcriptional responses to stimuli". In: *Molecular systems biology* 7.1 (2011), p. 529.

[10] Zihao Wang, Zhenquan Zhang, and Tianshou Zhou. "Analytical results for non-markovian models of bursty gene expression". In: *Physical Review E* 101.5 (2020), p. 052406.
